# Comparative Genomics of Thaumarchaeota From Deep-Sea Sponges Reveal Their Niche Adaptation

**DOI:** 10.3389/fmicb.2022.869834

**Published:** 2022-07-04

**Authors:** Peng Wang, Minchun Li, Liang Dong, Cheng Zhang, Wei Xie

**Affiliations:** ^1^School of Marine Sciences, Sun Yat-sen University, Zhuhai, China; ^2^School of Oceanography, Shanghai Jiao Tong University, Shanghai, China; ^3^Southern Marine Science and Engineering Guangdong Laboratory (Zhuhai), Zhuhai, China

**Keywords:** comparative genomics, deep-sea sponge, Thaumarchaeota, metagenomics, ammonia oxidation

## Abstract

Thaumarchaeota account for a large portion of microbial symbionts in deep-sea sponges and are even dominant in some cases. In this study, we investigated three new sponge-associated Thaumarchaeota from the deep West Pacific Ocean. Thaumarchaeota were found to be the most dominant phylum in this sponge by both prokaryotic 16S rRNA amplicons and metagenomic sequencing. Fifty-seven published Thaumarchaeota genomes from sponges and other habitats were included for genomic comparison. Similar to shallow sponge-associated Thaumarchaeota, those Thaumarchaeota in deep-sea sponges have extended genome sizes and lower coding density compared with their free-living lineages. Thaumarchaeota in deep-sea sponges were specifically enriched in genes related to stress adapting, symbiotic adhesion and stability, host–microbe interaction and protein transportation. The genes involved in defense mechanisms, such as the restriction-modification system, *clustered regularly interspaced short palindromic repeats (CRISPR)*/*Cas* system, and toxin-antitoxin system were commonly enriched in both shallow and deep sponge-associated Thaumarchaeota. Our study demonstrates the significant effects of both depth and symbiosis on forming genomic characteristics of Thaumarchaeota, and provides novel insights into their niche adaptation in deep-sea sponges.

## Introduction

The deep sea is one of the least explored ecosystems on earth. Despite the nonluminous and oligotrophic conditions with extreme hydrostatic pressure, the deep sea still harbors diverse taxa. Sponges (phylum Porifera), as benthic sessile invertebrates, account for high biodiversity and biomass in the benthic ocean (Cathalot et al., 2015; Grebmeier et al., 2015; Maldonado et al., 2017; Moitinho-Silva et al., 2017). Within the deep sea, the sponge habitat plays a crucial role in carbon and nitrogen cycling (Radax et al., 2012a; Kutti et al., 2013; Cathalot et al., 2015). Distributed in oceans and freshwater globally (Van Soest et al., 2012), these efficient filter feeders play an essential role in linking pelagic and benthic environments by consuming particulate organic matter and dissolved organic matter (Yahel et al., 2003; de Goeij et al., 2013).

Sponges often form stable and sometimes species-specific relationships with microbial symbionts (Taylor et al., 2007, 2013; Zhu et al., 2008; Podell et al., 2020), whose tissue volume can be comprised of up to 38% microbiome (Vacelet and Donadey, 1977). The symbiotic systems are at least partially sustained by metabolic exchange between hosts and symbionts, including nitrogen, carbon, and secondary metabolites (Taylor et al., 2007; Fan et al., 2012), as well as the assimilation and transformation of dissolved organic matter (de Goeij et al., 2013). The microbiome can help sponge hosts consume many metabolic waste products, like carbon and nitrogen compounds (Erwin and Thacker, 2008; Mohamed et al., 2008; Moeller et al., 2019; Rubin-Blum et al., 2019), providing secondary metabolites for predator and pathogen defense (Waters et al., 2014; Mori et al., 2018) and even contributing to the formation of the peripheral skeleton (Uriz et al., 2012). Sponge symbionts can be transferred horizontally and vertically (Steger et al., 2008; Björk et al., 2019), contributing to the possible long-term evolution of unique symbiotic strategies.

Phylogenetic analyses have shown that sponge symbionts often include a substantial amount of Archaea (Moeller et al., 2019) and are sometimes even dominated by Archaea (Preston et al., 1996). Most sponge archaeal symbionts have been identified as Thaumarchaeota, which are ammonia-oxidizing archaea (AOA; Moeller et al., 2019; Zhang et al., 2019; Haber et al., 2020). Thaumarchaeota have been found in various environments, including seawater (Walker et al., 2010; Santoro et al., 2015; Bayer et al., 2016; Ahlgren et al., 2017), estuarine regions (Xie et al., 2014, 2018), hot springs (Spang et al., 2012; Xie et al., 2015b), freshwater (Sauder et al., 2018), industrial wastewater (Li et al., 2016; Sauder et al., 2017), terrestrial soils (Lehtovirta-Morley et al., 2011; Tourna et al., 2011; Zhalnina et al., 2014; Xie et al., 2015a; Jung et al., 2016), marine sediments (Park et al., 2012; Xie et al., 2014), and sponges (Preston et al., 1996; Schleper et al., 1998; Hallam et al., 2006; Holmes and Blanch, 2007; Fan et al., 2012; Moitinho-Silva et al., 2017; Zhang et al., 2019). Some of those AOAs are sponge-specific (Simister et al., 2012). Moreover, Thaumarchaeota are more abundant in deep-sea sponges than in shallow-water sponges, highlighting the importance of the phylum for deep-sea sponges (Steinert et al., 2020).

The first genome for a Thaumarchaeota within a sponge (the demosponge *Axinella Mexicana*) belonged to *Cenarchaeum symbiosum* A (Hallam et al., 2006). Since then, a number of sponge-associated Thaumarchaeota metagenomes (Moitinho-Silva et al., 2017; Zhang et al., 2019; Haber et al., 2020), metatranscriptomes (Radax et al., 2012b; Moitinho-Silva et al., 2017), and metaproteomes (Moeller et al., 2019) have been obtained and analyzed. However, most studies have focused on basic descriptions of the genomes of sponge-associated Thaumarchaeota. Only a few studies have compared the genomes of sponge-associated Thaumarchaeota with those of their free-living counterparts (Moeller et al., 2019; Zhang et al., 2019; Haber et al., 2020). Comparative genomic analyses have revealed that some vital conserved genes, including genes involved in ammonia oxidization and vitamin synthesis, are shared among Thaumarchaeota from various environments. In contrast, genes associated with defense against phages, such as genes related to the restriction-modification (RM) system, toxin-antitoxin (TA) system and CRISPR/Cas system, are enriched in sponge-associated Thaumarchaeota. However, inconsistent results have been obtained regarding the characteristics of sponge-associated Thaumarchaeota. For instance, elevated GC contents in the genomes of sponge-associated Thaumarchaeota have been detected by Moeller et al. (2019) and Zhang et al. (2019) but not by Haber et al. (2020). A genome reduction in sponge-associated Thaumarchaeota has been reported in Zhang et al. (2019) but not in Haber et al. (2020) or Moeller et al. (2019). Importantly, the characteristics of deep-sea sponge-associated Thaumarchaeota (e.g., low GC content) differ from those of shallow-water sponges (Haber et al., 2020). However, the small number of deep-sea sponge-associated Thaumarchaeota genomes limits further analyses, particularly analyses of unique and shared strategies within deep-sea sponge-associated Thaumarchaeota.

In this study, three sponge-associated Thaumarchaeota metagenome-assembled genomes (MAGs) were separated from a deep-sea sponge collected at 2407.9 meters. Together with other 21 sponge-associated Thaumarchaeota and 28 free-living Thaumarchaeota (Supplementary Table S1) from both shallow water and deep sea, we expected to identify the unique genomic characteristics and symbiotic strategies within deep-sea sponge-associated Thaumarchaeota, as well as the pivotal and common mechanisms within all sponge-associated Thaumarchaeota. With three new Thaumarchaeota reported in this study, unique and ubiquitous properties of deep-sea sponge-associated Thaumarchaeota were systematically revealed through genomic comparison.

## Materials and Methods

### Sponge Sampling and DNA Extraction

A deep-sea sponge specimen was collected at a depth of 2407.9 meters in the Western Pacific (140.11548°E, 11.83154°N) near the Mariana Trench during the diving cruise SY219 on October 22, 2019. The manned submersible “Shen Hai Yong Shi” was used during the mission. The sponge sample SY219HM1 was cut into 1 cm^3^ pieces, stored in RNAlater, and immediately frozen at −80°C, as described previously (Feng et al., 2019). DNA was then extracted using the FastDNA SPIN Kit (MP Bio, Santa Ana, CA, United States) following the manufacturer’s protocol.

### DNA Sample Testing, Library Construction, and Metagenomic Sequencing

Two methods were used to test the quality of DNA samples: (i) DNA degradation degree and potential contamination was monitored on 1% agarose gels and (ii) DNA concentration was measured using Qubit® dsDNA Assay Kit in Qubit® 2.0 Flurometer (Life Technologies, CA, United States). DNA with OD value between 1.8 and 2.0 and contents above 1 μg was used to construct library.

A total amount of 1 μg DNA was used as input material for the DNA sample preparations. Sequencing library was generated using NEBNext® UltraTM DNA Library Prep Kit for Illumina (NEB, United States) following manufacturer’s recommendations and index codes were added to attribute sequences to the sample. Briefly, the DNA sample was fragmented by sonication to a size of 350 bp. The DNA fragments were end-polished, A-tailed, and ligated with the full-length adaptor for Illumina sequencing with further PCR amplification. At last, PCR products were purified (AMPure XP system) and the library was analyzed for size distribution by Agilent2100 Bioanalyzer.

The clustering of the index-coded sample was performed on a cBot Cluster Generation System according to the manufacturer’s instructions. After cluster generation, the library preparations were sequenced on an Illumina HiSeq platform and paired-end reads were generated.

### Metagenome Quality Control, Assembly, and Binning

A flexible pipeline, MetaWRAP version 1.2.1 (April 2019; Uritskiy et al., 2018), was used to process raw metagenomic reads into metagenomic assemblies and MAGs. After trimming using the metaWRAP-Read_qc module, the clean reads were then assembled using the metaWRAP-Assembly module with MegaHit version 1.1.3 (Grebmeier et al., 2015). Assemblies (contigs >2000 bp) were then binned using three binning methods in parallel to produce three initial bin sets of MAGs: CONCOCT version 1.0 (Alneberg et al., 2014), MaxBin2 version 2.2.6 (Wu et al., 2016), and metaBAT2 version 2.12.1 (Kang et al., 2015). Three initial bin sets were consolidated into a single bin set (consolidated bin set) using the Bin_refinement module, setting a minimum of 50% completion and maximum of 10% contamination, during which DAS_Tool (Sieber et al., 2018), Binning_refiner (Song and Thomas, 2017), and metaWRAP_Bin_refinment (Uritskiy et al., 2018) were used. Finally, bins in the consolidated bin set were reassembled using SPAdes version 3.13.0 (Bankevich et al., 2012) to improve their N50, completion, and contamination. The completion and contamination information of all MAGs including reference genomes were evaluated using CheckM version 1.1.3 (Parks et al., 2015).

### 16S rRNA PCR Amplification

The 16S rRNA of the deep-sea sponge sample was amplified by PCR using the universal primers for prokaryotes (F5′-GTGYCAGCMGCCGCGGTAA-3′; R 5′-GGACTACNVGGGTWTCTAAT-3′; Walters et al., 2016) with the PCR conditions: 30 s at 95°C, 30 s at 55°C, and 45 s at 72°C for 27 cycles. Amplicons were subjected to paired-end sequencing on the Illumina NovaSeq platform PE250 chemical at Novogene Bioinformatic Technology Co., Ltd. (Tianjin, China). The raw reads were deposited into the National Genomics Data Center (NGDC) Genome Sequence Archive (GSA) database (Accession Number: CRA006398). The raw 16S rRNA gene sequencing reads were quality-filtered by fastp version 0.20.0 (Chen et al., 2018) and merged by FLASH version 1.2.7 (Magoč and Salzberg, 2011). Operational taxonomic units (OTUs) with 97% similarity cutoff were clustered using UPARSE version 7.1 (Edgar, 2013), and chimeric sequences were identified and removed. The taxonomy of each OTU representative sequence was analyzed by RDP Classifier version 2.2 (Wang et al., 2007) against the 16S rRNA database (Silva v138) using confidence threshold of 0.7. A total of a total of 59,467 sequences with a mean length of 250 bp were retained. The coverage was greater than 99%, demonstrating the representation of those sequences.

### Taxonomic Annotation, Visualization, and Quantification of Assemblies and Quantification of MAGs

The metagenomic assemblies were annotated with their taxonomic assignments and bin membership information and projected onto GC vs. Abundance plots for visualization using Blobology version 1.0 (Kumar et al., 2013) and MegaBLAST version 2.6.0 (Morgulis et al., 2008). To quantify the abundance of each bin in the sample at the level of all reads, the metaWRAP-Quant_bins module (Uritskiy et al., 2018) was used. The process of quantification of bins was set before reassemble of bins based on the protocol of metaWRAP pipeline, therefore the consolidated bins were used in this process for more accurate result. Reassembled bins were used for later phylogenetic and comparative analyses as MAGs because reassembled bins have better genome quality after reassemble.

### Genome Dataset

Totally eight deep-sea sponge-associated Thaumarchaeota, 13 shallow-water sponge-associated Thaumarchaeota, eight shallow-water free-living Thaumarchaeota, 20 deep-sea free-living Thaumarchaeota, eight sentimental/soil Thaumarchaeota and two Aigarchaeota outgroup were included as referenced genomes in our study (Supplementary Table S1). All reference genomes are available in the NCBI/NGDC/JGI database.

### GTDB Classification and Phylogenetic Analysis of MAGs

GTDB-Tk version 1.3.0 (Chaumeil et al., 2019) was used to classify MAGs generated from this study as well as to build the phylogenetic tree with reference genomes. In phylogenetic analysis, 24 sponge-associated Thaumarchaeota, 28 free-living Thaumarchaeota, eight sediment/soil Thaumarchaeota and two Aigarchaeota outgroup were used (Supplementary Tables S4; S5). The classification and phylogenetic analysis of Thaumarchaeota were performed based on 122 archaeal marker genes (Supplementary Table S5), and 120 bacterial marker genes were used to classify bacterial MAG (Parks et al., 2018). GTDB-Tk classifies each genome based on its placement in the reference tree, its relative evolutionary divergence, and average nucleotide identity (ANI) to reference genomes based on GTDB database (Supplementary Table S4).

To build the phylogenetic tree, a multiple sequence alignment based on 122 archaeal marker genes was first created with GTDB-Tk align module. FastTree version 2.1.10 (Price et al., 2010) was used to infer the phylogenetic tree with ML Model based on the provided multiple sequence alignment. The local support value of each branch was calculated with Shimodaira-Hasegawa test based on default 1,000 resamples. The phylogenetic tree was drawn and presented using iTOL version 5 (Letunic and Bork, 2021) with the original tree document generated from GTDB-Tk.

The ANI values for sponge-symbiotic and free-living Thaumarchaeota genomes were calculated using FastANI version 1.32 (Jain et al., 2018), setting the minimum fragment length at 1500 bp.

### 16S rRNA Extraction and 16S Maximum-Likelihood Tree Construction

The 16S rRNA of each genome was extracted using Barrnap version 0.9 (Seemann, 2013). The extracted 16S rRNA genes were used to build Maximum-Likelihood Tree with Thaumarchaeota OTUs generated from 16S rRNA PCR amplification. The multiple sequence alignment was generated by MUSCLE version 3.8.1551 (Edgar, 2004). The IQ-TREE version 2.0.3 (Minh et al., 2020) was used to build maximum-likelihood tree based on the alignment. Tree was drawn and presented using iTOL version 5 (Letunic and Bork, 2021) with the treefile generated by IQ-TREE.

### Functional Annotation and Comparative Genomic Analyses

Three new sponge-symbiotic MAGs together with reference Thaumarchaeota genomes were annotated with Prokka version 1.13 (Seemann, 2014). Then, the clusters of orthologous groups (COGs; Tatusov et al., 2003) of each genome was annotated by Prokka based on database NCBI_nt v4 (Supplementary Table S8). The Kyoto Encyclopedia of Genes and Genomes (KEGGs) with E-value ≤0.01 of each Thaumarchaeota were annotated online with KofamKOALA ver. 2022-02-01 (KEGG release 101.0; Aramaki et al., 2019) using the faa file (FASTA Amino Acid file) generated from Prokka.

In order to identify predicted functions and genes (COG and KEGG) responsible for observed clustering patterns, a “random forest” algorithm was performed using R package Boruta version 7.0.0 (Kursa and Rudnicki, 2010; value of *p* < 0.05). Boruta tests if the importance of each individual variable is significantly higher that the importance of a random variable by fitting random forest models iteratively until all predictor variables are classified as “confirmed” or “rejected” at the 0.05 alpha level (Leutner et al., 2012). The “random forest” method were used in many relative studies in comparison of metagenomes (Costa et al., 2020; Tong et al., 2021) as well as genomes (Mageiros et al., 2021; Tong et al., 2021; Sawhney et al., 2022). Moreover, “random forest” selection was found to have the best predictive performance in genomic selection compared with other tree-based methods (Ashoori-Banaei et al., 2021). Heatmaps were drawn using pheatmap R package version 1.0.12 (Kolde, 2019). Only Thaumarchaeota with >95% genome completeness were used for predicting enriched/deprived COGs/KEGGs because low-completeness genomes can easily disturb the accuracy of comparison between number of predicted genes in two groups.

### Significant Difference Test

All significant tests were calculated using GraphPad Prism version 9.1.1 (233).^1^ Two-tailed unpaired *t*-tests were used for comparisons of genomic characteristics (i.e., GC content, genome size, and coding density) based on the data provided in Supplementary Tables S3, S7. If there are significant different variances between two groups, then two-tailed, unpaired Welch’s *t*-test was conducted instead.

Simple linear regression between genome size and coding density is calculated also using GraphPad Prism version 9.1.1 (233).

### Data Deposition

The whole genome sequence data, assemblies, and bins reported in this paper have been deposited in the NGDC (Chen et al., 2021; CNCB-NGDC Members and Partners, 2021), under BioProject Number PRJCA007290 at https://ngdc.cncb.ac.cn. Data for four MAGs and metagenomic assemblies can be accessed through Accession Numbers GWHBGCA00000000, GWHBGCB00000000, GWHBGBY00000000, GWHBGBZ00000000, and GWHBGBV00000000. Raw metagenome reads can be accessed through Accession Number CRA005418.

## Results

### Deep-Sea Sponge Sample and Metagenome Assemblies

A deep-sea sponge was collected at 2407.9 meters depth (Supplementary Figure S1). After DNA extraction and library construction, the metagenome of sponge was sequenced and 20.8 Gbps of raw metagenome reads were obtained. The raw reads were trimmed into pure reads, combined into contigs, and assembled into metagenomic assemblies. After removing contigs shorter than 1,000 bp, the metagenomic assemblies comprised 51,528 contigs with a total length of 107 Mb and the largest contig of 59,009 bp (Supplementary Figure S2). The N50 value was 2,179 bp and the L50 value was 13,756. The average GC content of our assemblies was 36.23%.

**Figure 1:**
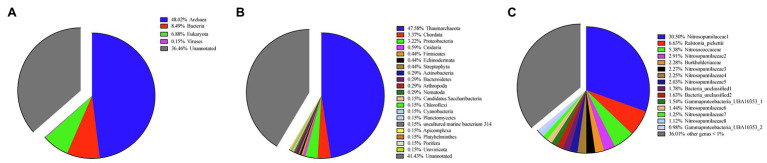
Taxonomy and quantification of metagenome assemblies at the **(A)** kingdom and **(B)** phylum level. **(C)** Taxonomy and quantification of 16S rRNA operational taxonomic units (OTUs) at family level.

Eukaryota (6.88% in all contigs), Bacteria (8.49% in all contigs), Archaea (48.02% in all contigs), and Viruses (0.15% in all contigs) genes were annotated in metagenomic assemblies (Figure 1; Supplementary Table S2). Within Eukaryota, the phyla Apicomplexa, Arthropoda, Chordata, Cnidaria, Echinodermata, Nematoda, Platyhelminthes, Porifera, and Streptophyta were annotated. Within contigs of Eukaryota, one contig was annotated as Porifera (Sponge) and its BLAST result showed the highest 93.83% identity with the partial 28S rRNA gene of a *Hertwigia* sp. *MD-2008* from Lyssacinosida order (E-value: 6e-23). Within Archaea, all annotated contigs were identified as Thaumarchaeota (47.58% in all contigs) at the phylum level. The high abundances of Thaumarchaeota by metagenomics were consistent with the amplicon sequencing results targeting prokaryotes 16S rRNA (Figure 1), which showed that nine Thaumarchaeotal OTUs (account for 43.7%) out of 15 OTUs higher than 1% were found in this sponge. Within Bacteria, the phyla Actinobacteria, Bacteroidetes, Candidatus Saccharibacteria, Chloroflexi, Cyanobacteria, Firmicutes, Planctomycetes, and Proteobacteria were annotated. The GC contents and abundances of contigs are shown in a scatter plot (Supplementary Figure S3). Eukaryota contigs had a wide range of GC contents but similar abundances, while Archaea contigs had similar GC contents but substantial variation in abundance. Bacteria contigs had relatively low abundances and contigs identified as Viruses were rare.

### General Summary of MAGs

Four MAGs with >50% completeness and <5% contamination were generated. These four MAGs, named bin1-bin4, were then reassembled to increase the genome quality. After reassembly, the completeness of bin1 and bin4 improved and contamination decreased for all bins (Supplementary Figures S4, S5). The proportion of each bin was quantified at the level of all reads (Table 1). As shown in Table 1, four bins accounted for about 37.0% of all assembled reads (bin4, 29.16%; bin1, 4.13%; bin2, 2.28%; bin3, 1.42%). The GC contents of bin1 (31.6%), bin2 (36.4%), and bin4 (33.1%) were lower than that of bacterial bin3 (54.3%). Within these four bins, the qualities of bin1 (size: 1.8 Mb, completeness: 88.83%, contamination: 0) and bin4 (1.3 Mb, 97.82%, 0.97%) were considered higher than those of bin2 (0.7 Mb, 53.87%, 0) and bin3 (0.9 Mb, 81.19%, 0), indicating near-completed genomes with little or no contamination (Table 1). The quality of contigs in bin2 was the lowest after reassembly, as evidenced by the lowest N50 value (5,963 bp; Supplementary Figure S4). All three other bins had high N50 values (>10,000 bp) after reassembly (Supplementary Figure S4).

**Table 1:** Metagenome-assembled genome properties and classification.

MAG name	Quant	CheckM complete	CheckM contam	GC (%)	ANI (%)	Strain	GTDB classification
bin.1	4.13	88.83	0	31.6	\	Unique	Archaea; Thermoproteota; Nitrososphaeria; and Nitrososphaerales
bin.2	2.28	53.87	0	36.4	98.05	Common	Archaea; Thermoproteota; Nitrososphaeria; and Nitrososphaerales
bin.3	1.42	81.19	0	54.3	\	Unique	Bacteria; Chloroflexota; Dehalococcoidia; and UBA1151
bin.4	29.16	97.82	0.97	33.1	95.42	Common	Archaea; Thermoproteota; Nitrososphaeria; and Nitrososphaerales

### Taxonomic Identity of Deep-Sea Sponge-Associated Thaumarchaeota

The reassembled bins were compared with reference bacterial or archaeal genomes for taxonomic classification based on Genome Database Taxonomy (GTDB; Supplementary Table S4). GTDB classification showed that bin1, bin2, and bin4 were members of the phylum Thermoproteota (also recognized as Thaumarchaeota), while bacterial bin3 belonged to the phylum Chloroflexi (Table 1). The ANI results against the GTDB database for bin1 and bin3 are not shown in Table 1 because the closest species with ANI ≥95% were not found within GTDB database. According to the Critical Assessment of Metagenomic Interpretation (CAMI) study (Sczyrba et al., 2017), “unique strains” were defined as genomes with <95% ANI compared with any other genome, while “common strains” were defined as genomes with an ANI ≥95%. Therefore, bin1 and bin3 were considered unique species based on GTDB database. The high abundance (35.6%, Table 1) of Thaumarchaeota bins within all assembled reads suggested its dominance within the deep-sea sponge. For the following analyses, we focused on Thaumarchaeota MAGs: bin1, bin2, and bin4.

### Phylogenetic Relationships and Genomic Similarity of Sponge-Associated and Free-Living Thaumarchaeota

**Figure 2:**
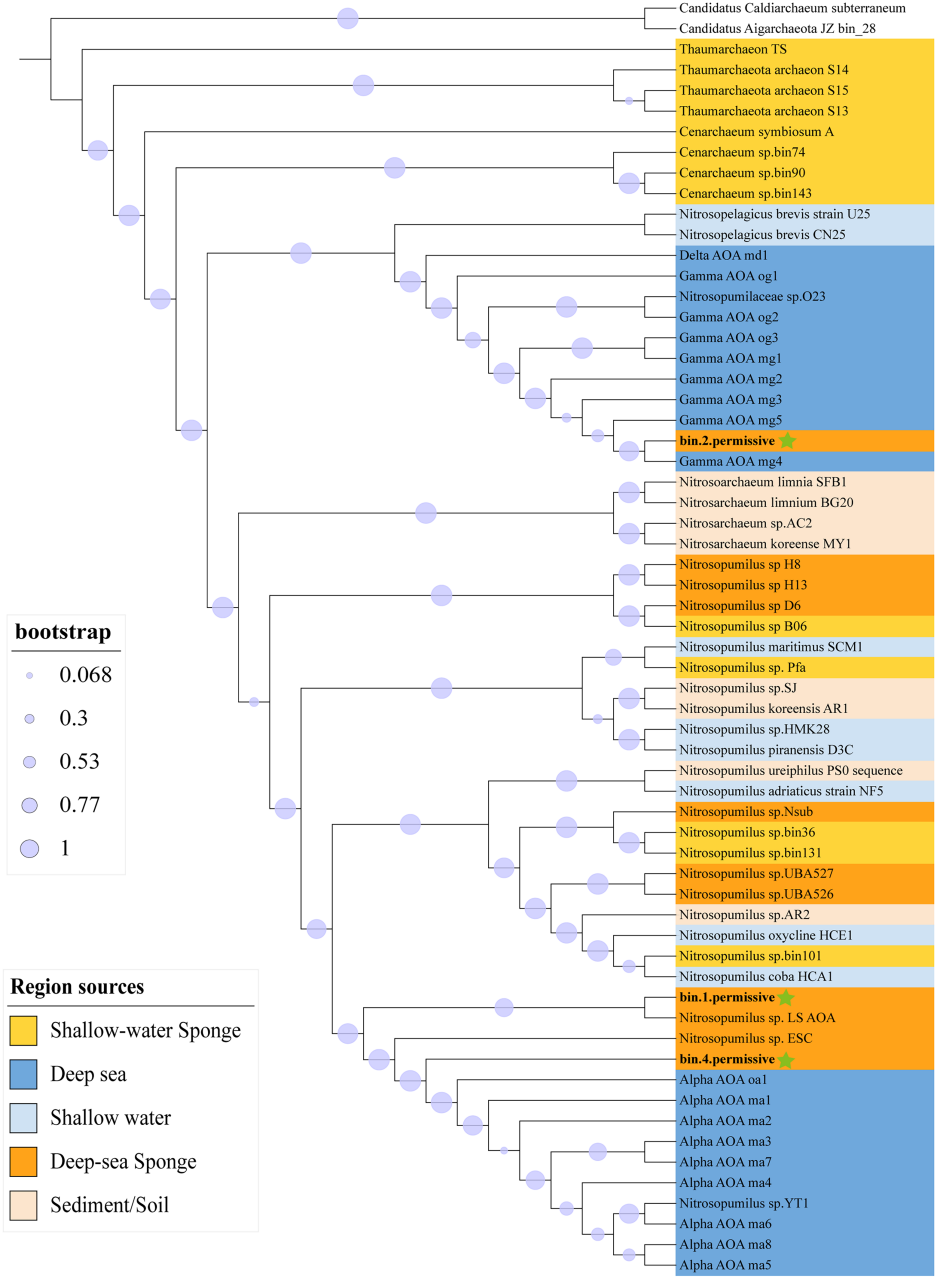
Maximum-likelihood GTDB-Tk phylogenetic tree of Thaumarchaeota based on 122 archaeal marker genes. The tree was rooted with two Aigarchaeota (*Candidatus Caldiarchaeum subterraneum*, and *Candidatus Aigarchaeota JZ bin_28*). The background color indicates the region sources of Thaumarchaeota. Three MAGs in this study are marked by green stars. Bootstrap values were calculated based on Shimodaira–Hasegawa test with 1,000 resamples.

Totally 60 Thaumarchaeota and two Aigarchaeota outgroups were aligned to 122 archaeal marker genes (Supplementary Table S5) then built a Maximum-Likelihood GTDB-Tk tree (Figure 2). Bin1 and bin4 were grouped within the deep-sea free-living Alpha Thaumarchaeota (Alpha AOA) lineage. Bin2, on the other hand, was assigned to the deep-sea free-living Gamma Thaumarchaeota (Gamma AOA) lineage. The deep-sea sponge-associated Thaumarchaeota were separated into four clades: Gamma AOA clade, Alpha AOA clade, sponge clade (i.e., H8, H13, and D6) and mixed clade (i.e., UBA526, UBA527, and Nsub). A relevant 16S rRNA tree was built based on the extracted 16S rRNA of genomes and 16S rRNA amplicon (Supplementary Figure S7). Two OTUs that accounted for 30.5% (OTU2) and 2.2% (OTU4921) were clustered together with bin1 and bin4’s 16S, demonstrating the representations of these two bins in the community.

The ANIs between all 60 Thaumarchaeota were calculated (Supplementary Table S6). All our three sponge-associated Thaumarchaeota bins were most closely related to the deep-sea free-living Thaumarchaeota. Bin1 and bin4 were most similar to the deep-sea Alpha Thaumarchaeota lineage while bin2 was most similar to the deep-sea Gamma lineage. Within sponge-associated Thaumarchaeota, bin1 (84.23%) and bin4 (87.87%) both had the highest ANI with *Nitrosopumilus_sp._ESC* recovered from deep-sea sponges. Overall, the ANI results were highly consistent with the phylogenetic tree based on 122 archaeal marker genes.

### Comparison of the Genomic Characteristics of Sponge-Associated and Free-Living Thaumarchaeota

We analyzed the GC contents of all Thaumarchaeota from sponges and the water column (based on Supplementary Table S3). The GC content of shallow-water sponge-associated Thaumarchaeota (mean = 47.12%, *N* = 13) was significantly greater (*p* = 0.0051, two-tailed, Welch’s *t*-test) than that of shallow-water free-living Thaumarchaeota (33.46%, *N* = 8). However, there was no significant difference (*p* = 0.0824, two-tailed, Welch’s *t*-test) between those of deep-sea sponge-associated Thaumarchaeota (39.45%, *N* = 11) and deep-sea free-living Thaumarchaeota (34.48%, *N* = 20).

**Figure 3:**
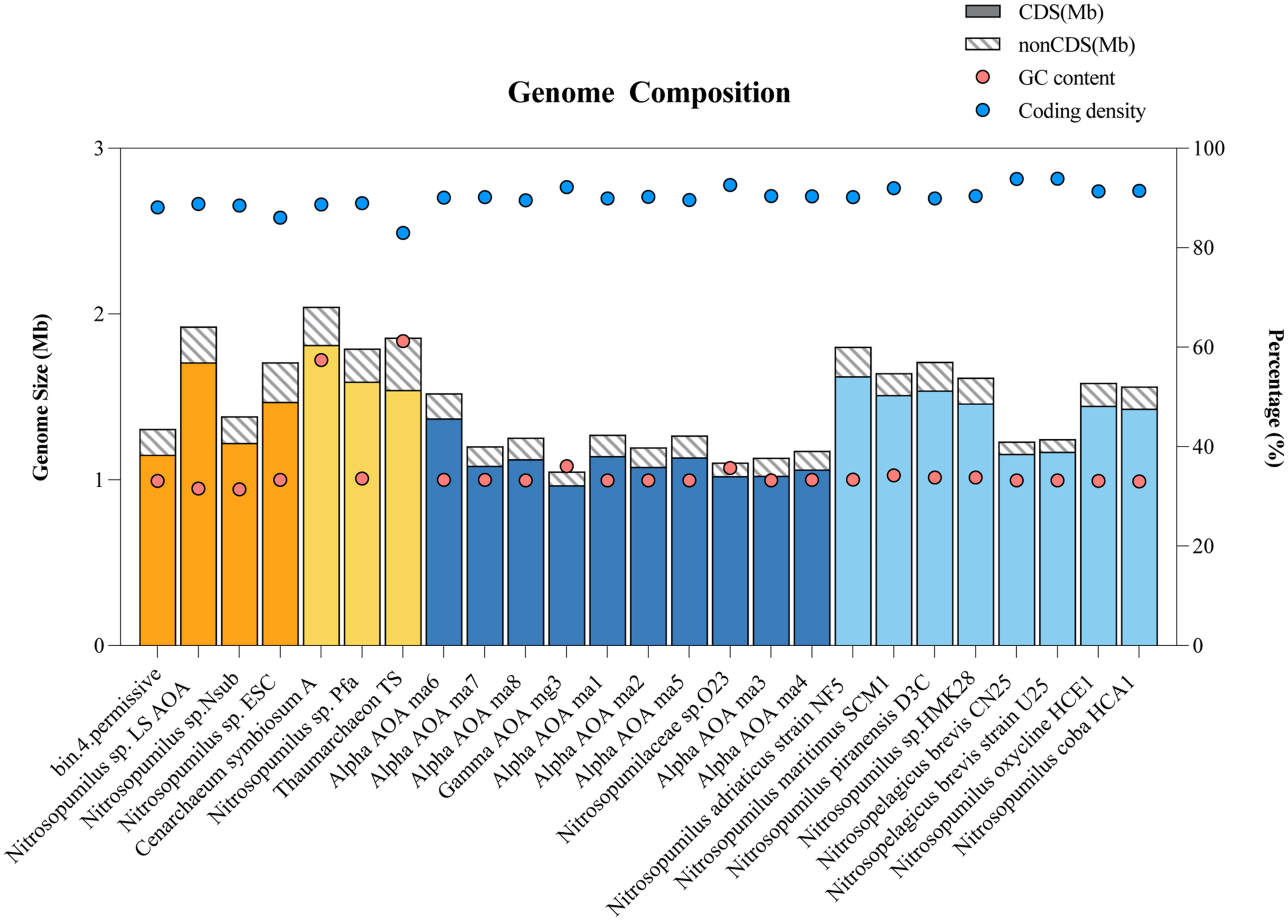
Bar chart and dot plot showing the genome composition of sponge-associated and free-living Thaumarchaeota with >95% completeness. Bar colors represent the location of Thaumarchaeota: Shallow-water sponge (yellow), Deep-sea sponge (orange), Shallow water (light blue), Deep sea (dark blue). Dots indicate the GC content (red) or coding density (blue). CDS, coding sequence and nonCDS, non-coding sequence.

The genome size, coding density, coding sequences (CDS), non-coding sequence (nonCDS), and GC contents of reference Thaumarchaeota with >95% completeness were shown in Figure 3. The genome size and coding density of Thaumarchaeota from sponges and the water column were compared (for genomes with completeness >95%, based on Supplementary Table S7). With respect to vertical distance, smaller genomes (*p* = 0.0007, two-tailed, unpaired *t*-test) were found in deep-sea free-living Thaumarchaeota (mean = 1.2 Mb, *N* = 10) than in shallow-water free-living Thaumarchaeota (1.5 Mb, *N* = 8). However, there was no significant difference (*p* = 0.1414, two-tailed, unpaired *t*-test) in genome size between deep-sea sponge-associated Thaumarchaeota (1.6 Mb, *N* = 4) and shallow-water sponge-associated Thaumarchaeota (1.9 Mb, *N* = 3). In a horizontal comparison, i.e., samples from the same depth but different habitat types, the genome size of deep-sea sponge-associated Thaumarchaeota (1.6 Mb, *N* = 4) was significantly larger (*p* = 0.0056, two-tailed, unpaired *t*-test) than that of deep-sea free-living Thaumarchaeota (1.2 Mb, *N* = 10). Similarly, the genome size of shallow-water sponge-associated Thaumarchaeota (1.9 Mb, *N* = 3) was significantly larger (*p* = 0.0256, two-tailed, unpaired *t*-test) than that of shallow-water free-living Thaumarchaeota (1.6 Mb, *N* = 8).

The coding density (Figure 3; Supplementary Table S7) of sponge-associated Thaumarchaeota was significantly lower than those of free-living Thaumarchaeota at all depths (3.6 ± 0.7%, *p* < 0.0001, two-tailed, unpaired *t*-test), shallow water (4.7 ± 1.4%, *p* = 0.0089, two-tailed, unpaired *t*-test), and deep sea (2.6 ± 0.6%, *p* = 0.0015, two-tailed, unpaired *t*-test). We fitted a linear regression between genome size and coding density for all Thaumarchaeota with >95% completeness and detected a negative relationship (simple linear regression, *R*^2^ = 0.25, *p* = 0.0111; Supplementary Figure S6).

### Functional Differences Between Sponge-Associated and Free-Living Thaumarchaeota

**Figure 4:**
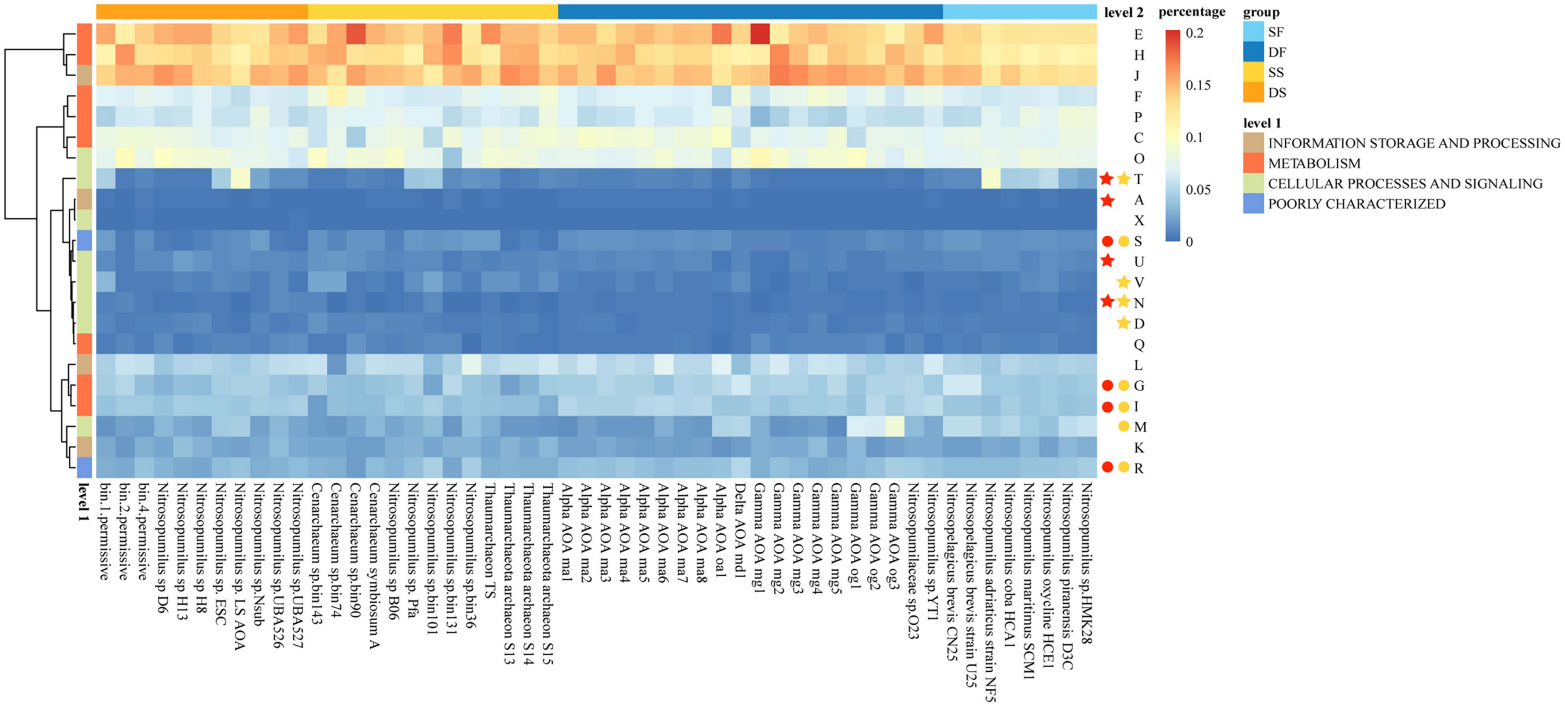
The percentages of clusters of orthologous group (COG) functional categories for sponge-associated and free-living Thaumarchaeota. Colors on top indicate the source: SF, shallow water (light blue); DF, deep sea (dark blue); SS, shallow-water sponge (yellow); and DS, deep-sea sponge (orange). Colors on the left indicate level 1 COG categories. The colored stars/circles after the level 2 functional categories indicate the relatively enriched/deprived functions in all sponge-associated Thaumarchaeota (yellow star/circle) and deep-sea sponge-associated Thaumarchaeota (red star/circle) based on random forest analyses.

Functional genes were predicted for sponge-associated and free-living Thaumarchaeota based on the COG database (Supplementary Table S8). We transformed gene counts in each COG functional category (level 2) into percentages and drew a heatmap (Figure 4). Boruta feature selection “random forest” analysis (*p* < 0.05) was used to identify the annotations that segregated significantly between sponge-associated and free-living Thaumarchaeota, or between deep-sea sponge-associated and deep-sea free-living Thaumarchaeota for their COG functional categories (Figure 4). Compared with those free-living Thaumarchaeota, the sponge-associated Thaumarchaeota were significantly enriched in functions for “Signal transduction mechanisms” (T), “Defense mechanisms” (V), “Cell motility” (N) and “Cell cycle control, cell division, chromosome partitioning” (D). Compared with those deep-sea free-living Thaumarchaeota, the deep-sea sponge-associated Thaumarchaeota were significantly enriched in functions for “Signal transduction mechanisms” (T), “RNA processing and modification” (A), “Intracellular trafficking, secretion, and vesicular transport” (U) and “Cell motility” (N). The results from KEGG annotation (level 2) showed that those deep-sea sponge-associated Thaumarchaeota were enriched in similar functions for “Signal transduction,” “Infectious disease: parasitic” and “Membrane transport” (Supplementary Table S9; Supplementary Figure S8), demonstrating the different characteristics of functional genes between sponge-associated Thaumarchaeota and their free-living relatives in the deep sea.

### Common and Unique Genes of Deep-Sea Sponge-Associated Thaumarchaeota

**Figure 5:**
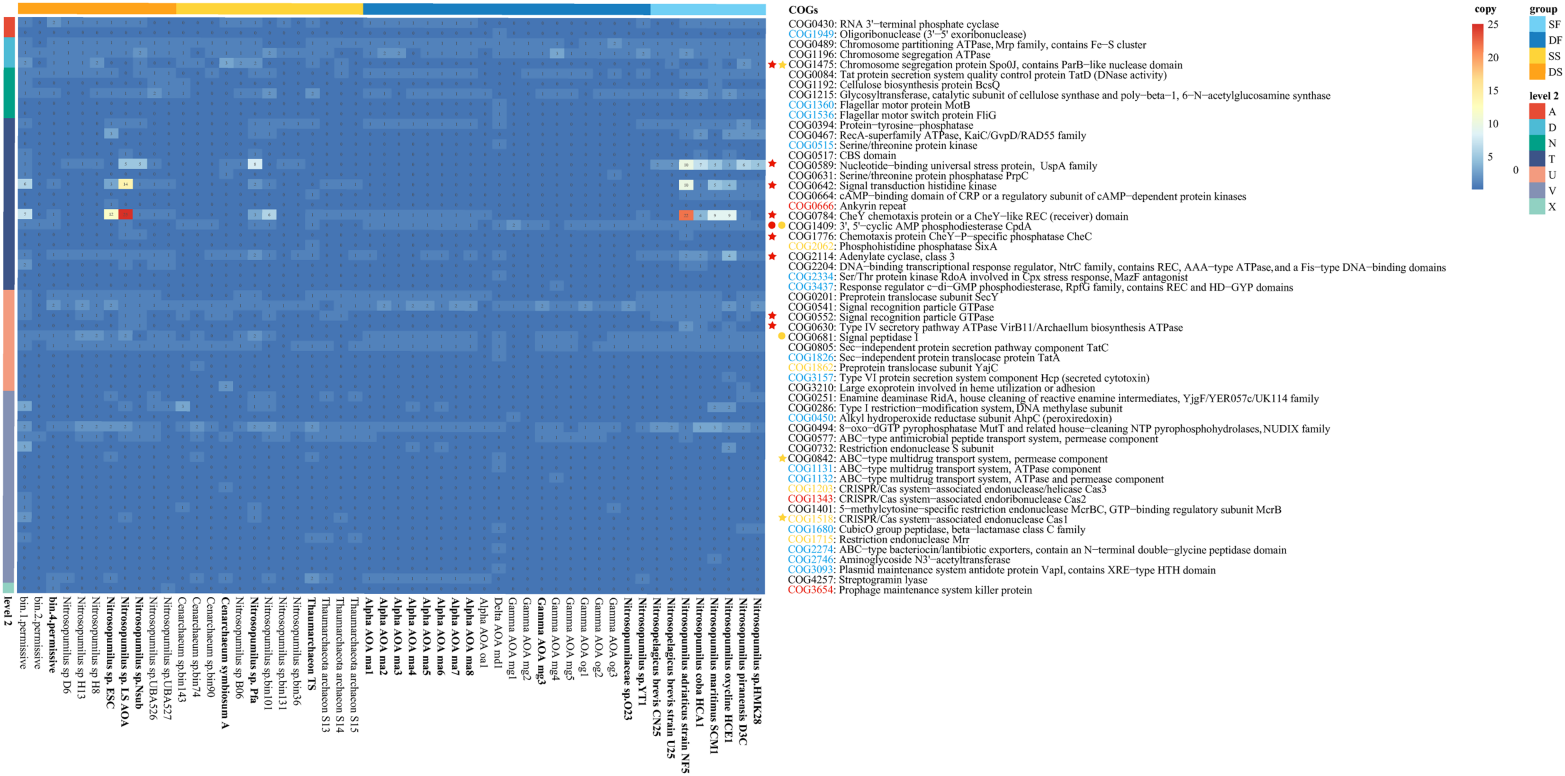
Gene copies of sponge-enriched COG functional categories of sponge-associated and free-living Thaumarchaeota. Gene numbers are shown with different colors. The color of each gene on the right shows its prevalence in sponge-associated Thaumarchaeota: red, only in sponge-associated Thaumarchaeota; blue, significantly higher in sponge-associated Thaumarchaeota. The colors on top indicate the source: SF, shallow water (light blue); DF, deep sea (dark blue); SS, shallow-water sponge (yellow); and DS, deep-sea sponge (orange). The colored stars/circles before the COGs indicate the relatively enriched/deprived functions in all sponge-associated Thaumarchaeota (yellow star/circle) and deep-sea sponge-associated Thaumarchaeota (red star/circle) with >95% completeness (genome name shown in bold text) based on random forest analyses. The color of COG number represents the COGs that were only annotated in sponge (yellow), deep-sea sponge (red) and water (blue).

Six sponge-enriched functions in COG level 2 categories (i.e., functions of A, D, N, T, U, and V), together with an additional function “Mobilome: prophages, transposons” (X) found only in deep-sea sponges, were further examined through presenting all possible COGs with at least one copy within all 52 Thaumarchaeota that have been annotated under these COG functions (Figure 5; Supplementary Table S8). The gene copy number of all 57 COGs for each Thaumarchaeota was presented in a heatmap (Figure 5). A total of 57 COGs that have at least one copy in either of those 25 Thaumarchaeota genomes with >95% completeness were included in the Boruta “random forest” comparative analysis. To figure out certain genes that contribute to the sponge-enriched functions, we checked those COGs in two dimensions: (i) COGs that had significantly higher numbers (based on 25 near-completed Thaumarchaeota) in all sponges (sponge-enriched COGs) or deep-sea sponges (deep-sea sponge-enriched COGs) compared to their relevant free-living counterparts and (ii) COGs that only presented in any of sponge-associated Thaumarchaeota (sponge-specific COGs) or any of deep-sea sponge-associated Thaumarchaeota (deep-sea sponge-specific COGs).

COGs that were only found in all sponge-associated Thaumarchaeota, deep-sea sponge-associated Thaumarchaeota and free-living Thaumarchaeota were shown in Figure 5. To sum up, there were a total of seven sponge-specific COGs (including three deep-sea sponge-specific COGs). Three deep-sea sponge-associated COGs were *Ankyrin repeat* (COG0666), *CRISPR/Cas system—associated endoribonuclease Cas2* (COG1343) and *Prophage maintenance system killer protein* (COG3654); while four other sponge-specific COGs were *Phosphohistidine phosphatase SixA* (COG2062), *Preprotein translocase subunit YajC* (COG1862), *CRISPR/Cas system—associated endonuclease/helicase Cas3* (COG1203) and *Restriction endonuclease Mrr* (COG1715).

Boruta “random forest” analysis was used to identify the COGs that were significantly different between sponge-associated and free-living Thaumarchaeota, or between deep-sea sponge-associated and deep-sea free-living Thaumarchaeota (Figure 5). In summary, three enriched COGs (COG1475, COG0842, and COG1518) and two deprived COGs (COG1409 and COG0681) were found in sponge-associated Thaumarchaeota compared to free-living Thaumarchaeota. As for deep-sea sponge-associated Thaumarchaeota, eight deep-sea sponge-enriched COGs (COG1475, COG0589, COG0642, COG0784, COG1776, COG2114, COG0552 and COG0630) and one deep-sea sponge-deprived COG (COG1409) were found compared to deep-sea free-living Thaumarchaeota.

Moreover, some sponge-enriched or sponge-specific genes found in COG annotation were also detected through KEGG annotation (Supplementary Table S10). *Chromosome segregation protein Spo0J, contains ParB—like nuclease domain* (COG1475) that enriched in both all sponges and deep-sea sponges was detected in KEGG annotation (K03497). The *3′, 5′—cyclic AMP phosphodiesterase CpdA* (COG1409) that deprived in both all sponges and deep-sea sponges was detected in KEGG annotation (K03651). Sponge-specific *Preprotein translocase subunit YajC* (COG1862) was only detected in sponge-associated Thaumarchaeota in KEGG annotation (K03210). However, the sponge-specific *Phosphohistidine phosphatase SixA* (COG2062) was not found in sponge-associated Thaumarchaeota but only in free-living Thaumarchaeota in KEGG annotation (K08296).

In general, more sponge-specific and sponge-enriched COGs were shown in deep-sea sponges than in all sponges compared to their free-living counterparts (Figure 5). COGs in “Signal transduction mechanisms” (T) were highly enriched or specific for deep-sea sponge-associated Thaumarchaeota. And the only gene, *Prophage maintenance system killer protein* (COG3654) in “Mobilome: prophages, transposons” (X) was exclusively annotated in deep-sea sponges (i.e., bin4 and UBA527). Deep-sea sponge-associated Thaumarchaeota were also highly enriched in *CheY chemotaxis protein or a CheY—like REC (receiver) domain* in both COG database (COG0784) and KEGG database (K03413, from map02030 “Bacterial chemotaxis”). The copies of COG0784 (mean = 9.75, *N* = 4) and map02030 (mean = 2.75, *N* = 4) in deep-sea sponge-associated Thaumarchaeota were higher than both deep-sea free-living Thaumarchaeota (COG0784: mean = 0.80, *p* = 0.21; map02030: mean = 0, *p* = 0.20; *N* = 10) and shallow-water sponge-associated Thaumarchaeota (COG0784: mean = 1.33, *p* = 0.24; map02030: mean = 0, *p* = 0.20; *N* = 3; Supplementary Figure S10). However, no significant difference (*p* < 0.05) was found for these comparisons based on unpaired two-tailed Welch’s *t*-test, which was possibly caused by the limited sponge-associated Thaumarchaeota genomes with >95% completeness. Those results demonstrated the importance of “Signal transduction mechanisms” (including Chemotaxis) for the deep-sea sponge-associated Thaumarchaeota. The only common result of sponge-enriched and deep-sea sponge-enriched COG was *Chromosome segregation protein Spo0J, contains ParB—like nuclease domain* (COG1475, K03497).

Similar to COG annotation, genes annotated based on KEGG also showed relevant enrichments of sponge-associated Thaumarchaeota in some frequently-discussed systems: CRISPR/Cas system, RM system and TA system (Supplementary Table S10). In summary, 37 genes in CRISPR/Cas system (with 35 sponge-specific genes), 94 genes in RM system (with 72 sponge-specific genes) and 12 genes in TA system (with 10 sponge-specific genes) were annotated in KEGG database. Some specific genes found in COG annotation were also found in KEGG annotation. For example, the *CRISPR/Cas2* of bin1 was found in COG annotation (COG1343) as well as KEGG annotation (K09951), but the CRISPR/Cas1 was not found in KEGG annotation.

## Discussion

### General Genomic Information for Deep-Sea Sponge-Associated Thaumarchaeota

In this study, the classification of both MAGs and 16S rRNA-gene amplicons showed the dominance of Thaumarchaeota within our deep-sea sponge (Figure 1). For metagenomic assemblies, the phylum within Archaea were all identified as Thaumarchaeota. For our assembled genomes, three of four MAGs reported in this study were classified to Thaumarchaeota (Table 1), which is consistent with previous studies (Moeller et al., 2019; Zhang et al., 2019; Haber et al., 2020). Moreover, the 16S rRNA analysis also showed that Thaumarcheotal OTUs account for 43.7% of all OTUs, which is consistent with previous 16S rRNA research that archaeal deep-sea sponge community structures were dominated by Thaumarchaeota (Steinert et al., 2020).

#### Genome Expansion

Differences in genome size between symbionts and free-living counterparts in the seawater column have been debated for a long time. A genome reduction was found in symbiotic bacteria and was attributed to adaptation within the host environment (McCutcheon and Moran, 2012; Zhang et al., 2018). Smaller genomes of sponge-associated Thaumarchaeota were found in a recent study by comparing with terrestrial and marine free-living Thaumarchaeota combined (Zhang et al., 2019). However, there was no size difference between sponge-associated Thaumarchaeota and marine free-living Thaumarchaeota when compared only with their marine counterpart. Moreover, no difference in genome size was found between sponge-associated Thaumarchaeota and free-living Thaumarchaeota in some studies (Moeller et al., 2019; Haber et al., 2020). Since marine Thaumarchaeota generally have smaller genomes than those of terrestrial Thaumarchaeota (Kellner et al., 2018; Qin et al., 2020), the genome reduction found by comparing sponge-associated Thaumarchaeota with non-oceanic Thaumarchaeota may be less convincing. In our study, smaller genomes were found in deep-sea free-living Thaumarchaeota compared to those of shallow-water free-living Thaumarchaeota. However, such a difference was not found between sponge-associated Thaumarchaeota from shallow water and deep sea. Such inconsistencies further demonstrated the importance of comparisons among sponge-associated and free-living Thaumarchaeota at the same depth.

Here, we focused on the comparison between sponge-associated Thaumarchaeota with oceanic free-living Thaumarchaeota at relevant depth. A significant genome expansion (*p* < 0.05) in sponge-associated Thaumarchaeota compared to free-living Thaumarchaeota was found both in deep sea and in shallow water. This genome expansion within both deep-sea and shallow-water sponge-associated Thaumarchaeota might indicate that the symbiotic strategies in shallow-water and deep-sea environment are similar. The gene content and organization may be influenced by both nutrient levels and habitat types. For example, Thaumarchaeota species at a neutral soil pH that occupy nutrient-enriched environments tend to have larger genomes and lower coding densities (Qin et al., 2020). Many bacterial symbionts within sponges contain several elements involved in horizontal gene transfer (Fan et al., 2012), suggesting that gene transfer between members of the sponge microbiome is common (Pita et al., 2018). More transposases were found in sponge-associated Thaumarchaeota than in free-living Thaumarchaeota (Moeller et al., 2019; Haber et al., 2020), suggesting that Thaumarchaeota in sponges could gain genes by horizontal transfer. Deep-sea and shallow-water sponges might provide Thaumarchaeota opportunities to gain larger genomes by repeatedly horizontal and vertical gene transfer.

#### Low Coding Density

Genome size can be influenced by both CDS and intergenic regions (non-CDS; Figure 3; Supplementary Table S7). Thaumarchaeota in a favorable environment tends to have a lower coding density than those in an unfavorable environment (Qin et al., 2020). Here, we found that sponge-associated Thaumarchaeota always showed significantly lower (*p* < 0.05) coding densities than those of free-living Thaumarchaeota, irrespective of depth. The lower coding density in sponge-associated Thaumarchaeota might be explained by the nutrient-rich conditions. Relatively enriched carbon and nitrogen compounds can be directly obtained from sponge hosts (Erwin and Thacker, 2008; Mohamed et al., 2008; Moeller et al., 2019; Rubin-Blum et al., 2019), providing sponge-associated Thaumarchaeota with adequate nutrients. Moreover, conditions in the deep sea may remain stable for hundreds of years. Such favorable conditions may promote long-term symbiotic relationships within deep-sea sponge hosts, resulting in a low coding density in deep-sea sponge-associated Thaumarchaeota.

Horizontal and vertical transmission of sponge bacterial and archaeal symbionts have been reported (Björk et al., 2019). Importantly, vertical transmission of sponge Thaumarchaeota was found by fluorescence *in situ* hybridization (Steger et al., 2008). Therefore, a healthy and stable environment may enable the maintenance of such genome of lower coding density. At the same time, the vertical transmission of Thaumarchaeota within sponge may provide opportunities for Thaumarchaeota with low coding densities to retain important characteristics across generations. With the appropriate environment to gain a larger yet less compact genome and the required mechanism for vertical transmission, Thaumarchaeota within deep-sea and shallow-water sponges could gradually evolve larger genomes with lower coding densities during the long-term symbiotic relationship.

A large-scale comparative analysis of Thaumarchaeota worldwide has shown an apparent linear decrease in coding density with genome size (Qin et al., 2020). We observed a similar pattern but with a weaker correlation coefficient (Supplementary Figure S6), which might be explained by variance among sponge-associated Thaumarchaeota.

#### Low GC Content

GC enrichment has been observed in some obligate bacterial symbionts compared with their free-living counterparts (Slaby et al., 2017). Some previous research has shown that shallow-water sponge-associated Thaumarchaeota generally have higher GC contents than those of free-living terrestrial Thaumarchaeota (Moeller et al., 2019; Zhang et al., 2019). However, the pattern investigated from the comparison between different environments (i.e., marine sponge and terrestrial environment) might be inappropriate. Moreover, the pattern of shallow-water sponges might not be appropriate for deep-sea sponges for their different characteristics. For example, the GC contents of deep-sea sponge-associated Thaumarchaeota generally show a low GC content based on recent studies (Bayer et al., 2020; Haber et al., 2020). This pattern is further supported by our results showing that deep-sea sponge-associated Thaumarchaeota genomes have a low GC content (31.6%–36.4%). In our genomic comparison, GC enrichment was only found in shallow-water sponge-associated Thaumarchaeota and not in deep-sea sponge-associated Thaumarchaeota compared to their free-living counterparts, which is consistent with a previous study (Haber et al., 2020). The mechanism underlying GC enrichment in the shallow-water sponges was not ubiquitous, and the pattern in deep-sea sponge-associated Thaumarchaeota requires further investigation.

The similar results of shallow-water and deep-sea sponge-associated Thaumarchaeota in genome expansion and lower coding density might indicate similar symbiotic strategies shared among Thaumarchaeota to live within sponge hosts regardless of depth. However, the limited number of sponge-associated Thaumarchaeota genomes, especially high-quality genomes, made the comparison less convincing. Therefore, more high-quality sponge-associated Thaumarchaeota genomes are still on the call and these patterns for sponge-associated Thaumarchaeota require further investigation.

### Functions Specifically Enriched in Deep-Sea Sponge-Associated Thaumarchaeota

In COG functional categories, the function of “Signal transduction mechanisms” (T) was enriched in both all sponges and deep-sea sponges (*p* < 0.05). At the gene level (level 3), five deep-sea sponge-enriched COGs (*p* < 0.05), one deep-sea sponge-specific COG and one sponge-specific COG accounted for the enrichment of the function “Signal transduction mechanisms”. However, all five deep-sea sponge-enriched COGs were not enriched anymore when considering shallow-water Thaumarchaeota genomes (sponge and free water) together. This might be due to the high copies of these COGs in shallow-water free-living Thaumarchaeota that undermine the presence of these COGs detected within shallow-water sponge-associated Thaumarchaeota. Moreover, the difference might suggest the unique characteristics and adapting mechanisms of Thaumarchaeota within deep-sea sponges.

#### Stress Adapting

In this study, *Nucleotide—binding universal stress protein, UspA family* (named UspA, COG0589) was found significantly enriched in deep-sea sponge-associated Thaumarchaeota compared to deep-sea free-living Thaumarchaeota. The conserved universal stress proteins (USPs) are found in bacteria, archaea, fungi, plants and even invertebrates animals (Vollmer and Bark, 2018). USPs are classified into UspFG-like proteins that can bind ATP and UspA-like proteins that cannot bind ATP (Tkaczuk et al., 2013). The universal stress protein UspA is thought to function as a general responder under various stresses such as oxidative stress, heat shock, low pH, hypoxia, nutrient starvation, and DNA-damaging agents threatening (O'Toole et al., 2003; O'Connor and McClean, 2017). Thaumarchaeota in deep-sea sponges could constantly face various stresses, such as invading phages, fluctuating nutrient conditions, and current disturbances (shear forces) from constantly filtering (Hadas et al., 2006; Jahn et al., 2019). Moreover, sponges can pump up to 24,000 liters of seawater through their system per day, exposing them up to an estimated ∼2.4 × 10^13^ viruses daily (Weisz et al., 2008). Therefore, Thaumarchaeota in deep-sea sponges might therefore encounter higher stress from shear forces and phages compared with their free-living relatives. The enriched genes related to UspA might help deep-sea sponge-associated Thaumarchaeota adapt those stresses.

#### Symbiotic Adhision and Stability

In this study, genes realted to two-component signal transduction systems (TCST systems) were specifically enriched in deep-sea sponge-associated Thaumarchaeota compared to deep-sea free-living Thaumarchaeota, which were *Signal transduction histidine kinase* (COG0642), *CheY chemotaxis protein or a CheY—like REC (receiver) domain* (COG0784 and K03413), and *Chemotaxis protein CheY*—*P—specific phosphatase CheC* (COG1776). TCST systems play major roles in the process of a variety of biological processes (Beier and Gross, 2006). In TCST systems, histidine kinases (HKs) play major roles in signal transduction by sensing stimulus and transmitting to response regulators (RRs), which in most cases bind to DNA and mediate cellular responses (Bhate et al., 2015). The *Signal transduction histidine kinase* (COG0642) found in this study is a kind of HK, while CheY (COG0784) and CheC (COG1776) were genes related to RR. Moreover, CheY and related “Bacterial Chemotaxis” pathway (KEGG map02030) were found higher in deep-sea sponge-associated Thaumarchaeota compared to Thaumarchaeota from shallow-water sponges and deep-sea (Supplementary Figure S10). A given bacterium can have tens to hundreds of different TCST systems (Ulrich and Zhulin, 2010) that allow it to sense and adapt to a variety of environmental signals such as osmotic changes (Tomomori et al., 1999), temperature (Albanesi et al., 2009), small molecules (Kaspar et al., 1999) and antimicrobials (Miller et al., 1989). In *H. parasuis* strain, CheY was proved to play an extremely important role in biofilms formation and autoagglutination through *cheY* gene deletion (He et al., 2016). Therefore, the deep-sea sponge-enriched CheY and CheC might also help Thaumarchaeota with biofilm formation and autoagglutination within deep-sea sponge hosts, which might be essential for Thaumarchaeota to resist share forces from host pumping and interact with symbiotic communities within deep-sea sponges.

Previous study has found several genomes of sponge microbiome contained gene families encoding adhesive molecules and type IV pilus structures, which were expected to counteract the strong shear foreces from high seawater flow rate induced by sponge pumping (Podell et al., 2020). In our study, Type IV secretory pathway ATPase VirB11/Archaellum biosynthesis ATPase (named FlaI, COG0630) was found specifically enriched within deep-sea sponge-associated Thaumarchaeota compared to deep-sea free-living Thaumarchaeota. The archaella (the pilus stucture in archaea) are widespread and have been found to be involved in swimming mobility in archaea (Ghosh and Albers, 2011; Jarrell and Albers, 2012). FlaI, an ATPase (Ghosh et al., 2011), is one of the six conserved subunits encoded by all archaella loci (Banerjee et al., 2012). Enriched FlaI of sponge-associated Thaumarchaeota was not previously described in deep-sea sponge-associated Thaumarchaeota. We consider it might associate with stronger swimming mobility in deep-sea sponge-associated Thaumarchaeota to counteract the constant current disturbance from the pumping of deep-sea sponge host, while the environments of free-living deep-sea Thaumarchaeota are usually relatively stable.

#### Host–Microbe Interactions

In our study, the only *Ankyrin repeat* (COG0666) was annotated in *Nitrosopumilus* sp. *ESC* from a deep-sea sponge. Ankyrin repeat (ANK) proteins constitute one of the most abundant families of repeat proteins, usually implicated in specific protein–protein interactions (Islam et al., 2018). They are involved in a diverse set of cellular processes, such as signal transduction, cell cycle regulation, vesicular trafficking, transcriptional regulation and more (Mosavi et al., 2004). ANKs were found in all forms of life, including archaea (Islam et al., 2018). The numerous genes encoding ANK-containing proteins found in the genome of various pathogenic bacteria and eukaryotic viruses suggest such genes were acquired from eukaryotes through horizontal gene transfer (Bork, 1993; Al-Khodor et al., 2010). Noteworthily, ANK repeat proteins were found remarkably enriched in obligate intracellular bacteria compared to other bacteria, which further suggests that ANK repeats are signature features of eukaryotic proteins and supports that intracellular bacteria widely use ANK repeats to interact with the eukaryotic host cell (Bork, 1993). Many bacterial pathogens can use various types of secretion systems to deliver ANK-containing proteins, therefore they can enter eukaryotic cells and mimic or manipulate various host functions (Al-Khodor et al., 2010). Therefore, the ANK found in *Nitrosopumilus sp. ESC* might be necessary for its host–microbe interaction and might even grant it the ability to enter and manipulate deep-sea sponge host cells.

#### Protein Transportation

In this study, *Signal recognition particle GTPase* (named FtsY, COG0552) was found as deep-sea sponge-enriched COG. The signal recognition particle (SRP) is a ribonucleoprotein complex involved in the targeting of nascent proteins to translocation sites contained in Eukarya, Bacteria and Archaea (Althoff et al., 1994; Rapoport et al., 1996; Stroud and Walter, 1999). SRP plays a critical role in the cotranslational targeting of proteins to the plasma membrane of prokaryotes (Saraogi and Shan, 2011). FtsY is the SRP receptor (Luirink et al., 1994), which can interact with ribosome-nascent-chain complex and therefore guide it to the protein-conducting channel in the plasma membrane (Valent et al., 1998). The SRP pathway in Archaea represents an intermediate between the bacterial and eukaryal systems (Pohlschröder et al., 1997; Eichler, 2000). Significant enriched FtsY found in deep-sea sponge-associated Thaumarchaeota might suggest higher efficiency and stronger demand of deep-sea sponge-associated Thaumarchaeota for cotranslational targeting in protein transportation.

This speculation could be further supported by our result that the only *Preprotein translocase subunit YajC* (named YajC, COG1862, K03210) was detected in a shallow-water sponge-associated Thaumarchaeota (i.e., bin74). YajC complex could transiently interact with the translocon, assisting the processes of posttranslational protein translocation and cotranslational integration of membrane proteins (du Plessis et al., 2011). However, such sponge-specific YajC was found only in one shallow-water sponge-associated Thaumarchaeota without significant difference. Therefore, further investigation of the process of protein transportation in sponge-associated Thaumarchaeota will be needed.

### Functions Commonly Enriched in Both Shallow and Deep Sponge-Associated Thaumarchaeota

In our study, genes in sponge-associated Thaumarchaeota were significantly enriched for the function of “Defense mechanisms” (V) at COG level 2 compared to genes in free-living Thaumarchaeota (*p* < 0.05). We found the enrichments of those common-shared genes were involved in CRISPR-Cas system, RM system, and TA system in all sponge-associated Thaumarchaeota, suggesting that these functions have vital and common roles for their adaptation to the hosts.

#### Phage Defense Mechanisms and Programmed Cell Death

Sponge symbionts are likely to be exposed to numerous kinds of phages due to the high filtration activity of their hosts (Hadas et al., 2006; Weisz et al., 2008; Jahn et al., 2019). Some sponges might even host individual and species-specific viral communities (Jahn et al., 2019). Both sponge metagenomes and sponge-associated bacterial genomes have been reported to be enriched for genes involved in defense against phages, such as phage growth limitation systems, RM system, TA system, and CRISPR/Cas systems (Fan et al., 2012; Horn et al., 2016; Slaby et al., 2017; Podell et al., 2019; Haber et al., 2020). These frequently discussed mechanisms were found again in our study.

CRISPR and CRISPR-associated (Cas) genes are often more abundant in the genomes of sponge-associated bacterial symbionts than in their free-living counterparts (Fan et al., 2012; Horn et al., 2016), and such patterns have also been found in sponge-associated Thaumarchaeota (Moeller et al., 2019; Haber et al., 2020). This was further supported by our finding that all three kinds of CRISPR/Cas genes [i.e., *CRISPR/Cas1* (COG1518), *CRISPR/Cas2* (COG1343, K09951) and *CRISPR/Cas3* (COG1203, K07012)] were only detected in sponge-associated Thaumarchaeota in this study. Moreover, *CRISPR/Cas2* was only detected in bin1 in our analysis, while *Cenarchaeum symbiosum A* was the only Thaumarchaeota genome that encoded *CRISPR/Cas3*. CRISPR/Cas protein families function in the immune system in many prokaryotic genomes, providing cell immunity against phage infection and foreign DNA expression (Bolotin et al., 2005; Haft et al., 2005). Sponge symbionts are exposed to a large amount of foreign DNA due to the high filtration rate of the host (Hadas et al., 2006; Van Soest et al., 2012; Jahn et al., 2019). Therefore, sponge-associated Thaumarchaeota might need genes, such as CRISPR/Cas, to defend against invading phages and survive in the symbiotic environment. Our findings further established the significant role of the CRISPR-Cas system in the symbiotic relationship between Thaumarchaeota and the sponge host.

Enrichment for RM systems has recently been found in many bacterial symbionts within sponges (Burgsdorf et al., 2015; Horn et al., 2016; Slaby et al., 2017). Additionally, the enrichment of RM systems in some sponge-associated Thaumarchaeota has previously been described (Moeller et al., 2019; Zhang et al., 2019; Haber et al., 2020). In our study, restriction enzymes, including endonucleases and methylases, were enriched or were only detected in sponge-associated genomes. Bin1 and three shallow-water sponge-associated Thaumarchaeota were the only Thaumarchaeota harboring the restriction endonuclease *Mrr* (COG1715). Moreover, the unique Thaumarchaeota bin1 also harbored three copies of *Type 1 restriction-modification system* (COG0286) and *Restriction endonuclease S subunit* (COG0732). The high abundance and uniqueness of these genes suggested that RM systems are important for Thaumarchaeota in sponges. RM systems represent a primitive immune system in bacteria. They are broadly found in prokaryotes due to their role in defense against invading DNA and in additional cellular processes, thereby increasing survival rates (Vasu and Nagaraja, 2013). Therefore, large quantities of genes in the RM system emphasize the need for sponge-associated Thaumarchaeota to defend against invading DNA, such as phages. Moreover, such genes in the RM system might be beneficial for Thaumarchaeota survival within the complicated symbiotic community. Increased copies of genes in the RM system in bin1 might reflect its long-term symbiotic history and successful adaptation within the deep-sea sponge.

Genes in TA systems have been found almost exclusively in sponge bacterial symbionts, rather than free-living bacteria (Slaby et al., 2017). Components of several TA modules have been found in sponge-associated Thaumarchaeota, and some modules are even uniquely found in sponge-associated Thaumarchaeota (Moeller et al., 2019; Haber et al., 2020). Interestingly, bin4 of our study included the only gene related to function “Mobilome: prophages, transposons” (X) among all Thaumarchaeota evaluated, i.e., *Prophage maintenance system killer protein* (COG3654, named Doc). Doc belongs to the type II TA system and could kill host cells (Altermann et al., 2005). Moreover, another protein, Phd, which is coexpressed with Doc, could counteract this killer protein. The lack of Phd could cause cell death and is usually induced upon phage or plasmid curing (Lehnherr et al., 1993). Doc acts asa lethal protein, while Phd is the antidote. TA systems found in most prokaryote genomes are composed of a toxin protein and a counteracting antitoxin and are thought to play essential roles in phage defense, stress responses, and programmed cell death (Song and Wood, 2020). Therefore, the only copy of the killer protein found in bin4 might function in phage defense under constant exposure to phages from seawater. This killer protein in the TA system might also help bin4 balance the symbiotic system within the deep-sea sponge host *via* the manipulation of host cell death.

Bin2 in our study showed no genes involved in these systems. This might be explained by the incomplete genome of bin2 (53.87%). More high-quality sponge-associated Thaumarchaeota genomes are needed in the future to detect genes related to phage defense, the regulation of host cell death, and adaptation to the symbiotic community.

#### Adaptation Under Catabolite Repression

Within the function of “Signal transduction mechanisms,” *3′, 5′—cyclic AMP phosphodiesterase CpdA* (COG1409) was the only sponge-deprived COG found in both deep-sea sponges and all sponges. The regulation of intracellular concentration of cAMP is dependent not only on the synthesis of cAMP but also on the hydrolysis of cAMP by cAMP phosphodiesterase (Imamura et al., 1996). In low glucose conditions, cAMP can act as an intracellular signaling molecule that allows bacteria to adapt to this changing environment and use a secondary source of carbon such as lactose, such process is known as catabolite repression (Bhadra et al., 2021). Low glucose conditions could activate adenylyl cyclase which increases the cAMP concentration, and through a series of processes, relevant enzymes and transport proteins were expressed for the usage of available carbon sources (Ullmann and Monod, 1968; Rickemberg, 1974; De Crombrugghe et al., 1984). Therefore, sponge-associated Thaumarchaeota might have lower copies of cAMP phosphodiesterase, which leads to higher cAMP concentration, to help them better utilize the available carbon sources in low glucose conditions within the sponge host. In the initial stage of adaption within the sponge, Thaumarchaeota might undergo similar catabolite repression because they face a changing environment due to the horizontal transfer from free water to sponge hosts.

Moreover, *Adenylate cyclase, class 3* (COG2114) was found enriched in deep-sea sponge-associated Thaumarchaeota compared to deep-sea free-living Thaumarchaeota. Class III adenylate cyclases are signaling proteins present in bacteria, archaea, and eukaryotes. This cAMP-producing enzymes were reported to be able to translate diverse intracellular and extracellular stimuli into a uniform intracellular signal (Bassler et al., 2018). Therefore, enriched adenylate cyclase might also lead to higher cAMP concentration which might again lead to a better adaptation within deep-sea sponges under potential catabolite repression.

#### Specific Chromosome Partitioning Mechanism

In our study, *Chromosome segregation protein Spo0J, contains ParB—like nuclease domain* (named Spo0J, COG1475, K03497) was considered as both sponge-specific and deep-sea sponge-specific COG, showing the importance of this gene in all sponge-associated Thaumarchaeota. Low-copy-number genomes like bacterial chromosomes and certain plasmids have evolved partitioning (Par) mechanisms to ensure that daughter cells receive a full complement of the genetic material, in contrast to high-copy-number plasmids that rely on random partitioning (Khare et al., 2004). ParB is one of the three required components in all chromosomal or plasmid Par systems in prokaryotes (Møller-Jensen et al., 2000). ParB, as a DNA-binding protein that recognizes and bins specifically to the centromere-like site (Mori et al., 1989), can interact with an ATPase (ParA) whose activity is essential for partitioning (Motallebi-Veshareh et al., 1990; Watanabe et al., 1992; Davis et al., 1996). Spo0J is a member of the ParB protein families, which is required for accurate chromosome partitioning (Ireton et al., 1994). Therefore, the Spo0J found in sponge-associated Thaumarchaeota might also be essential for the accurate chromosome partitioning within sponges. Constantly facing invading phages together with constant shear forces from host pumping, Thaumarchaeota in sponges might have stronger needs for chromosome partitioning mechanism than free-living Thaumarchaeota.

### Niches and Evolution of Sponge-Associated Thaumarchaeota in the Deep Sea

The deep-sea sponge-associated Thaumarchaeota formed four separate deep-sea Thaumarchaeota clusters, instead of a single cluster (Figure 2). This is in line with previous results (Moeller et al., 2019; Zhang et al., 2019; Haber et al., 2020), suggesting that the depth is an important determinant factor for the evolution of Thaumarchaeota. Moreover, some of those deep-sea sponge-associated Thaumarchaeota (e.g., bin1, LS AOA, and ESC) clustered differently with deep-sea free-living Thaumarchaeota in genomic functional categories (Supplementary Figure S8) and phylogenetic tree (Figure 2). These COG analyses also showed the distinct metabolic profiles between sponge-associated and free-living Thaumarchaeota (Supplementary Figure S8), suggesting that the symbiosis relationship was also a significant determinant factor for the evolution of Thaumarchaeota. Another interesting result is that our three newly reported sponge-associated Thaumarchaeota showed different enriched genes and clustered dispersedly in genomic functional categories (Supplementary Figure S8), which is consistent with former reports (Moeller et al., 2019; Zhang et al., 2019; Haber et al., 2020), suggesting the diverse adaptation strategies of those sponge-associated Thaumarchaeota that might be caused by the different ecological niches in sponges.

## Conclusion

Our results revealed that Thaumarchaeota species were dominant in a sponge from the deep Western Pacific Ocean. Three novel Thaumarchaeota genomes were found in this sponge. Together with other 57 published Thaumarchaeota genomes from sponges for comparison, we found those deep-sea sponge-associated Thaumarchaeota had larger genomes and lower coding densities than their deep-sea free-living lineages. Based on genomic comparison analyses, deep-sea sponge-associated Thaumarchaeota uniquely showed enriched or specific genes related to signal transduction mechanisms and intracellular trafficking mechanisms, such as universal stress protein UspA (stress adapting), chemotaxis protein CheY (biofilm formation), archaellum (swimming ability) and ankyrin repeat (host-microbe interation), suggesting the unique genomic characteristics and adapting mechanisms of Thaumarchaeota within deep-sea sponges. Such enrichments might be important for their adaption under various stresses, such as shear forces from host pumping, catabolite repression and invading phages in deep-sea sponges. Genes involved in defense mechanisms and symbiotic relationships, such as genes related to the RM systems (defense invading DNA and symbionts fitting), CRISPR/Cas systems (defense phages), and TA system (defense phages and manipulate host cell death), were highly enriched in both shallow and deep-sea sponge-associated Thaumarchaeota, suggesting the pivotal and common roles of those genes for the adaptations of those Thaumarchaeota to the hosts. The high enrichment of genes involved in defense mechanisms indicated that those sponge-associated Thaumarchaeota species continuously face invading phages within sponges. Moreover, different enriched genes and dispersed clustering of different sponge-associated Thaumarchaeota suggest that taxa are associated with different ecological niches and evolutionary stages within sponges.

## Data Availability Statement

The data presented in the study are deposited in the NGDC repository, accession number GWHBGCA00000000, GWHBGCB00000000, GWHBGBY00000000, GWHBGBZ00000000, GWHBGBV00000000, CRA006398, and CRA005418. Available at NGDC from China National Center for Bioinformation (CNCB): https://ngdc.cncb.ac.cn/search/?dbId=gwh&q=GWHBGBV00000000.

## Author Contributions

WX and LD designed research. WX, PW, ML, and CZ performed research. WX and PW analyzed data and wrote the paper. All authors contributed to the article and approved the submitted version.

## Funding

This work was supported by the National Natural Science Foundation of China [grant nos. 92051117 (WX), 41776137 (WX), and 42072332 (LD)], Southern Marine Science and Engineering Guangdong Laboratory (Zhuhai) [SML311019006 and SML311020006], and the Project of China Geological Survey [DD20191002 (WX)].

## Conflict of Interest

The authors declare that the research was conducted in the absence of any commercial or financial relationships that could be construed as a potential conflict of interest.

## Publisher’s Note

All claims expressed in this article are solely those of the authors and do not necessarily represent those of their affiliated organizations, or those of the publisher, the editors and the reviewers. Any product that may be evaluated in this article, or claim that may be made by its manufacturer, is not guaranteed or endorsed by the publisher.

## Footnotes

^1^ https://www.graphpad.com/
