# Supplementary material for: Comparative Genomics of Thaumarchaeota From Deep-Sea Sponges Reveal Their Niche Adaptation
Source: Front Microbiol. 2022 Jul 4;13:869834. doi: 10.3389/fmicb.2022.869834 (PMC9289680; doi:10.3389/fmicb.2022.869834)
Supplement: Supplementary file 1 [file Data_Sheet_1.pdf]

Supplementary figures

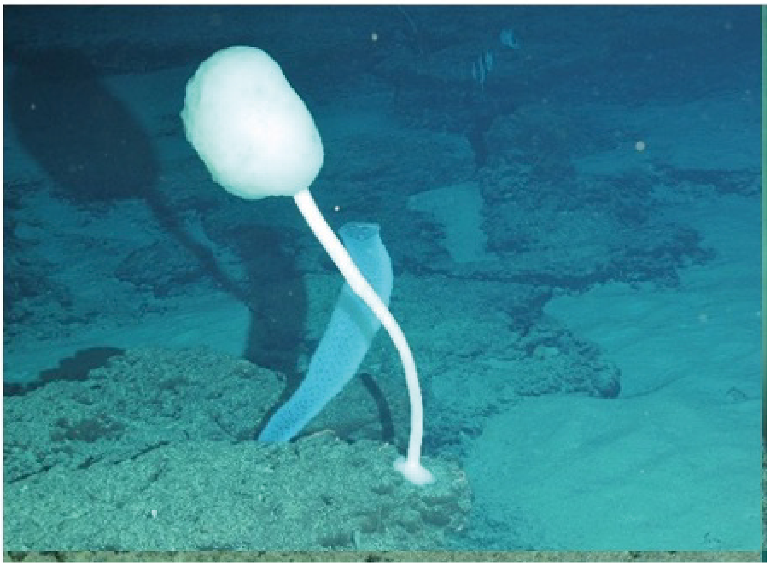

Figure S1.The in-situ photo of the deep-sea sponge in our study. The "ball" structure of the sponge in the front was sampled.

	final assembly
# contigs (>= 0 bp)	51528
# contigs (>= 1000 bp)	51528
# contigs (>= 5000 bp)	2307
# contigs (>= 10000 bp)	405
# contigs (>= 25000 bp)	25
# contigs (>= 50000 bp)	1
Total length (>= 0 bp)	107035809
Total length (>= 1000 bp)	107035809
Total length (>= 5000 bp)	18511781
Total length (>= 10000 bp)	6039367
Total length (>= 25000 bp)	886261
Total length (>= 50000 bp)	59009
# contigs	51528
Largest contig	59009
Total length	107035809
GC (%)	36.23
N50	2179
N75	1441
L50	13756
L75	29045
# N's per 100 kbp	0.00

Figure S2. Assemblies information of sponge metagenome, only contigs ≥ 1000 bp were remained in assemblies.

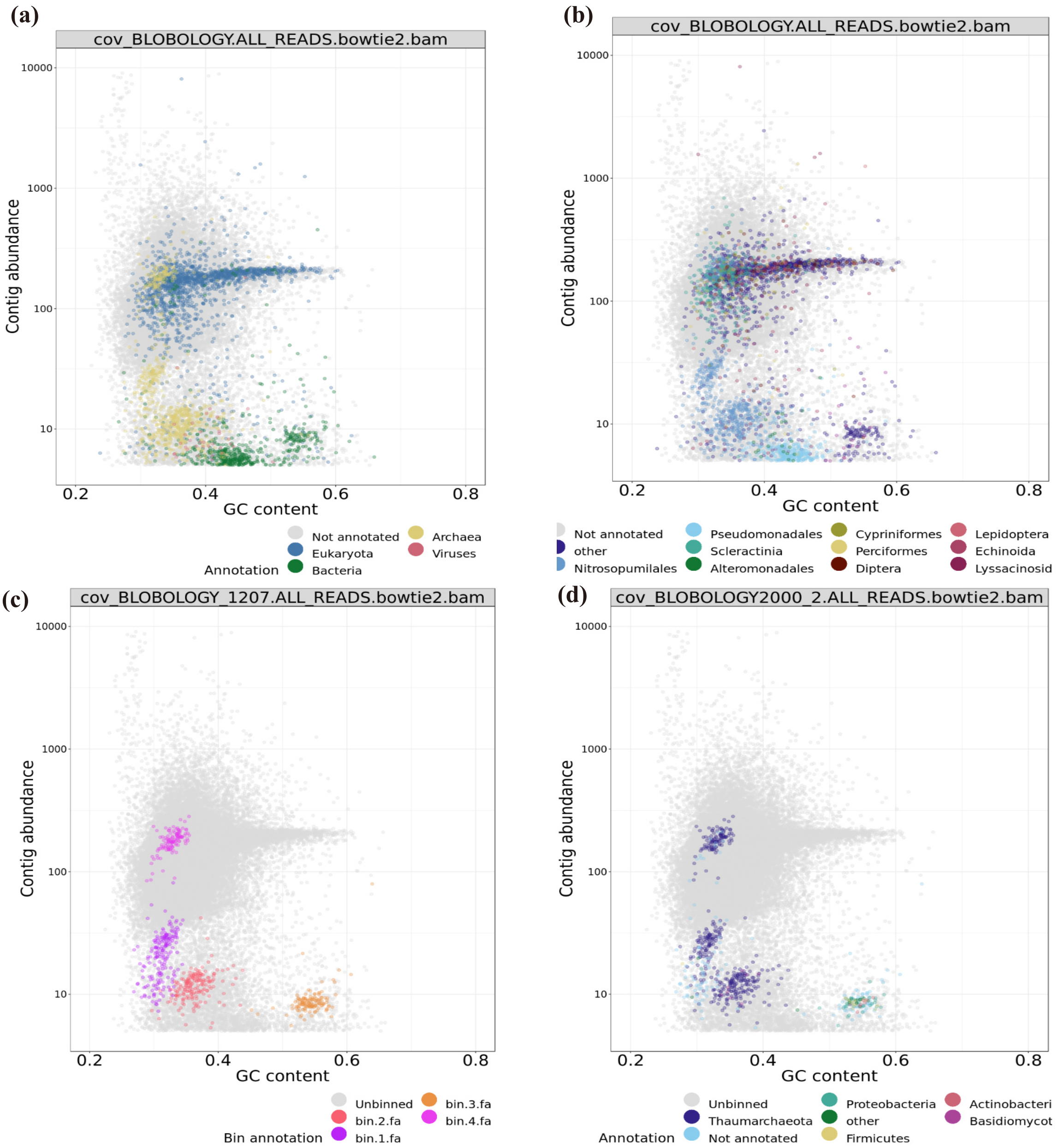

Figure S3. The GC vs Abundance plot of contigs of sponge metagenome by Blobology. The grey dots represented the contigs that could not be annotated. (a) Superkingdom level. (b) Phylum level. (c) MAGs that successfully binned. (d) Phylum level within bins.

Reassemble	bin	completeness	contamination	GC	lineage	N50	size	binner
Before	bin.4	97.08	0.97	0.33	Archaea	15938	1280331	binsA
	bin.1	88.83	0.323	0.315	Archaea	15570	1836444	binsA
	bin.3	81.18	0	0.543	Bacteria	8384	914803	binsBC
	bin.2	55.32	1.941	0.363	Archaea	3911	698300	binsBC
After	bin.4.permissive	97.81	0.97	0.33	Archaea	20817	1307109	/
	bin.2.permissive	53.87	0	0.363	Archaea	5963	670425	/
	bin.1.permissive	88.83	0	0.315	Archaea	30232	1838395	/
	bin.3.permissive	81.18	0	0.543	Bacteria	11605	918540	/

Figure S4. The information of sponge MAGs before and after reassembled. The suffix “orig” means original, “permissive” means better genomic quality after reassembled. The binner represent the source of different bin sets: binsA, metaBAT2; binsB, MaxBin2; binsAB, both metaBAT2 and MaxBin2.

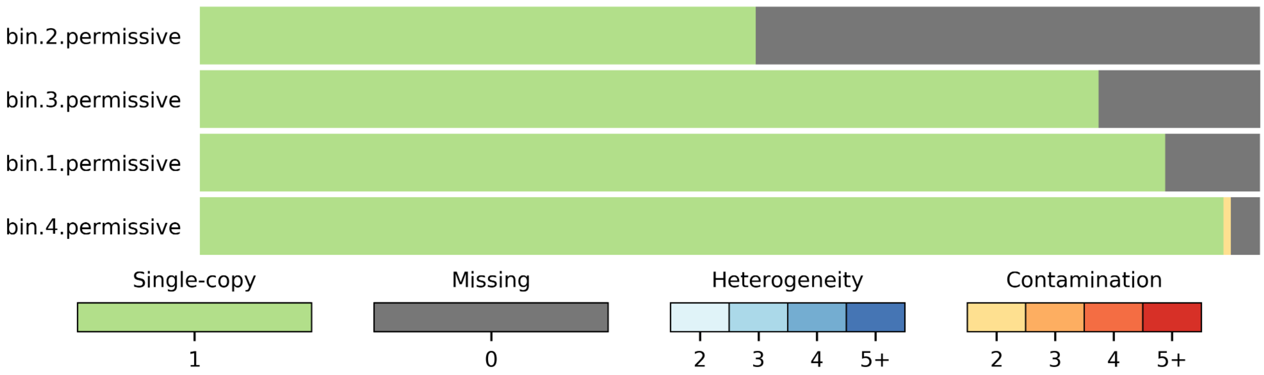

Figure S5. The completeness and contamination of reassembled bins. Heterogeneity represents the contamination from closely related taxa.

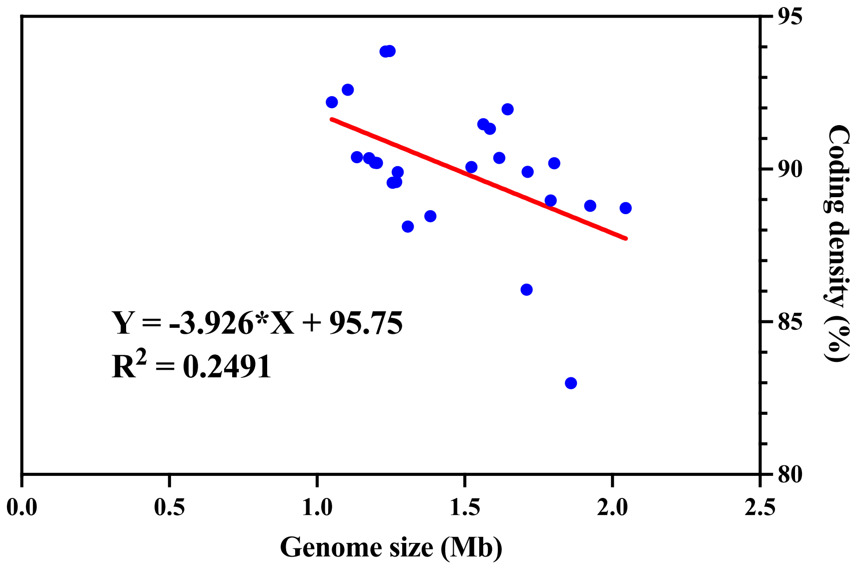

Figure S6. Linear regression model of Coding density and Genome size of all AOA > 95 % completeness, Y = -3.926*X + 95.75 (p = 0.0111).

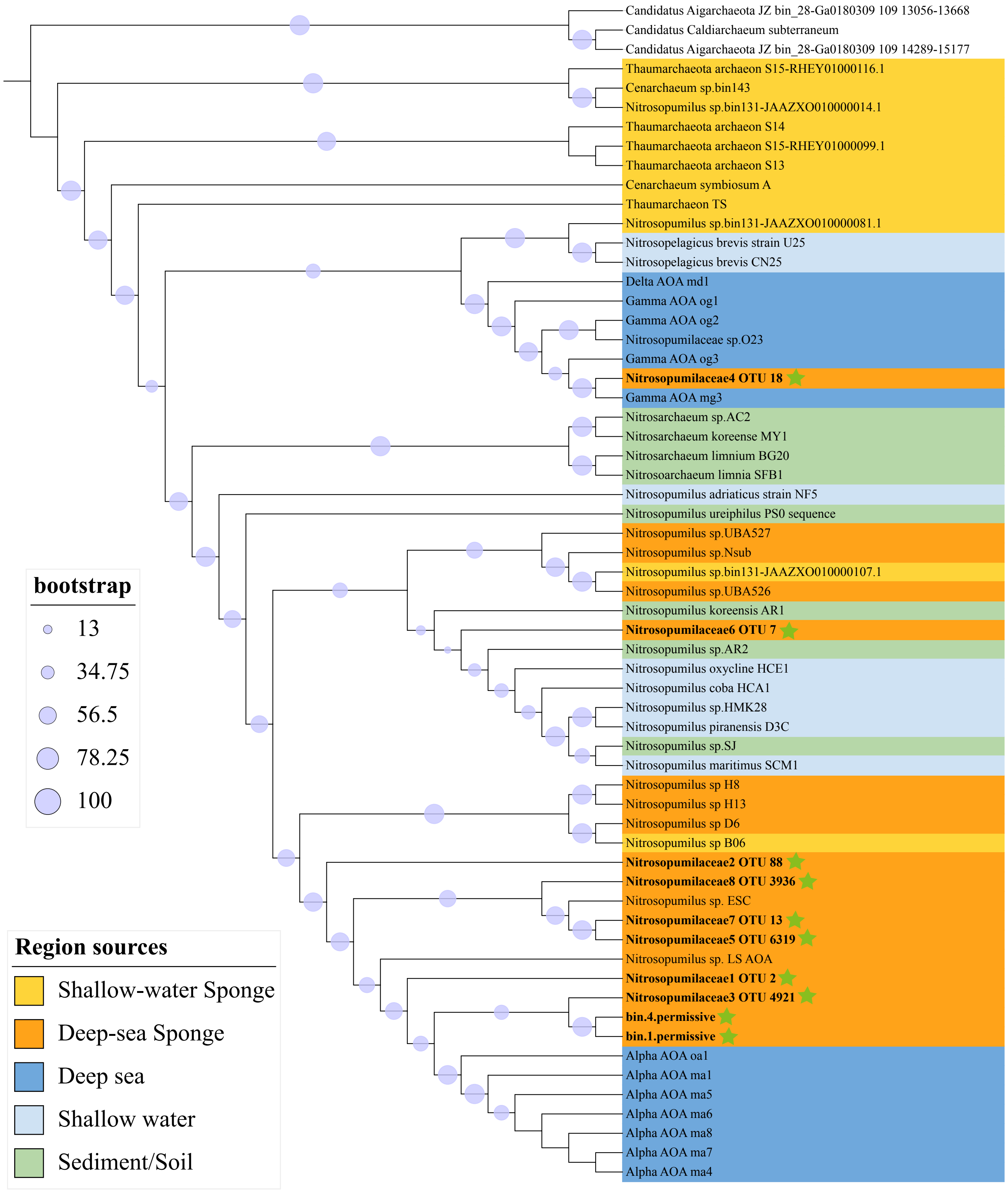

Figure S7. Maximum-likelihood 16S rRNA tree of Thaumarchaeota based on extracted 16S rRNA sequence and 16S rRNA amplicon (suffix OTU). The genomes with multiple 16S rRNA extracted were named with specific sequence fragment name in the suffix. The tree was rooted with three Aigarchaeota extracted 16S rRNA sequence (*Candidatus Caldiarchaeum subterraneum* and *Candidatus Aigarchaeota JZ bin_28*). The background color indicates the source of Thaumarchaeota. MAGs and OTUs reported in this study are marked by green stars with bold text. Bootstrap values were calculated based on 1000 repeats.

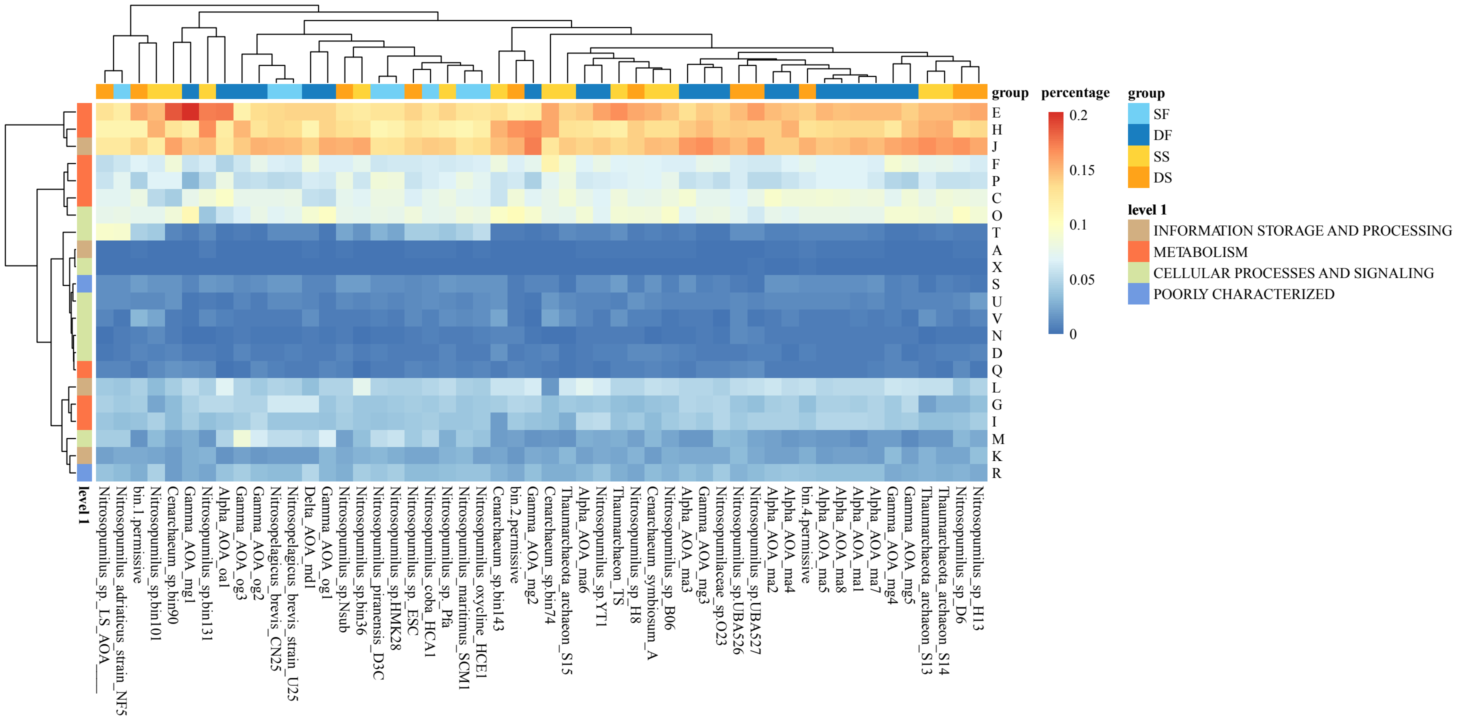

Figure S8. The percentages of COG level 2 functional categories for sponge-associated and free-living Thaumarchaeota. Both level 2 categories and Thaumarchaeota genomes were clustered. Colors on top indicate the source: SF, shallow water (light blue); DF, deep sea (dark blue); SS, shallow-water sponge (yellow); DS, deep-sea sponge (orange). Colors on the left indicate level 1 COG categories.

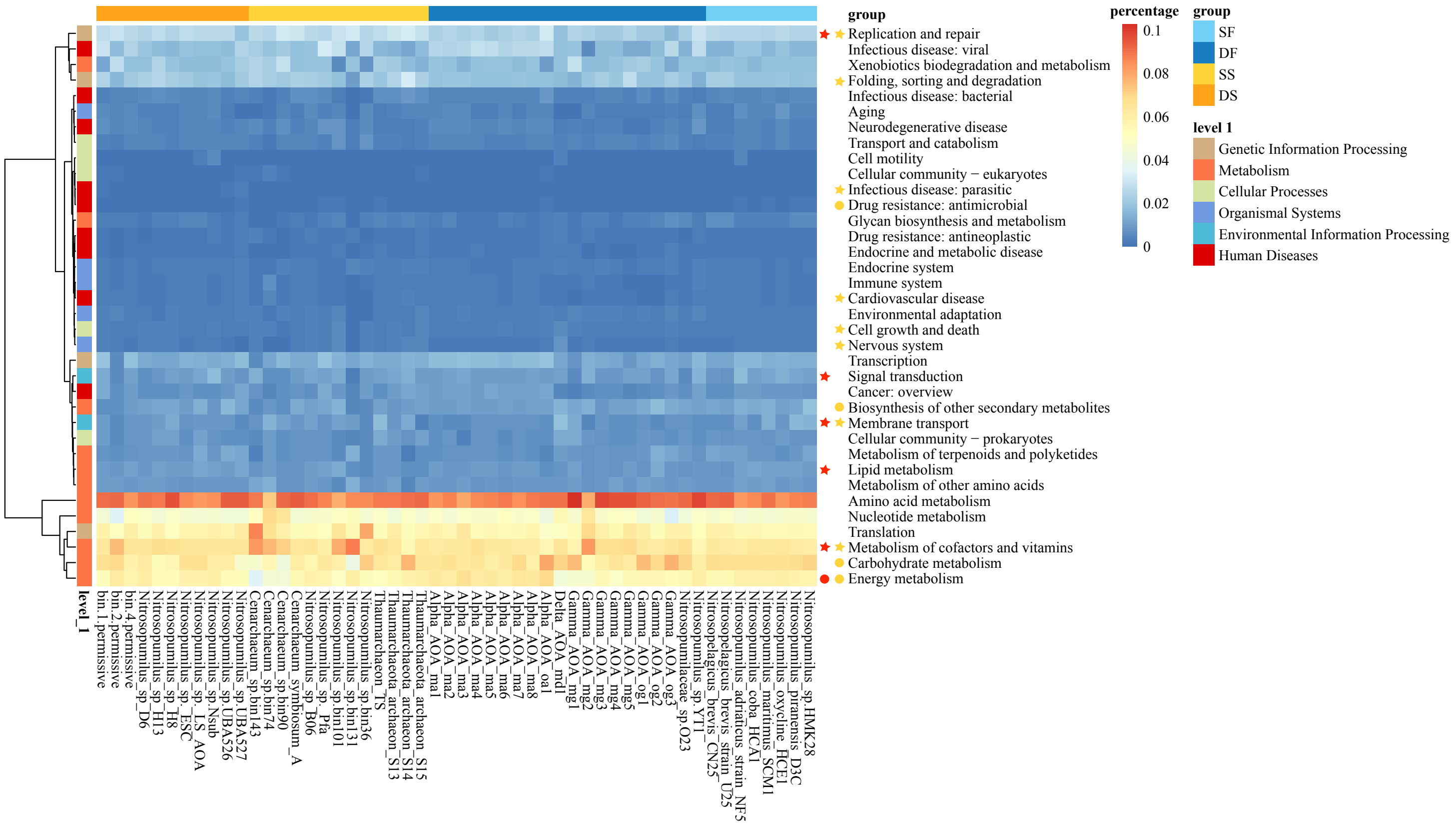

Figure S9. The percentages of KEGG level 2 functional categories for sponge-associated and free-living Thaumarchaeota. Colors on top indicate the source: SF, shallow water (light blue); DF, deep sea (dark blue); SS, shallow-water sponge (yellow); DS, deep-sea sponge (orange). Colors on the left indicate level 1 KEGG categories. The colored stars/circles before the level 2 functional categories indicate the relatively enriched/deprived functions in all sponge-associated Thaumarchaeota (yellow star/circle) and deep-sea sponge-associated Thaumarchaeota (red star/circle) based on random forest analyses.

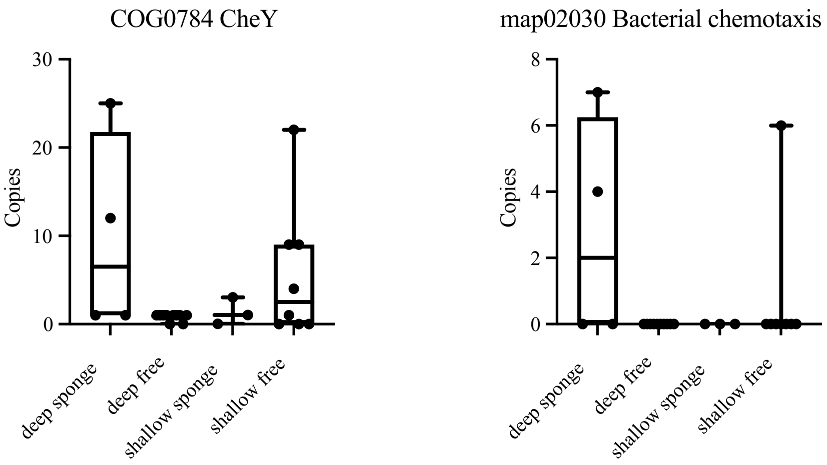

Figure S10. The boxplots of the number of related CheY genes or pathway annotated in COG (left) and KEGG (right) databases.
